# Supplementary material for: Metabolic programs define dysfunctional immune responses in severe COVID-19 patients
Source: Cell Rep. 2021 Feb 26;34(11):108863. doi: 10.1016/j.celrep.2021.108863 (PMC7908880; doi:10.1016/j.celrep.2021.108863)
Supplement: Document S1. Tables S1–S3 and Figures S1–S7 [file mmc1.pdf]

Cell Reports, Volume *34*

Supplemental information

Metabolic programs define dysfunctional immune responses in severe COVID-19 patients

Elizabeth A. Thompson, Katherine Cascino, Alvaro A. Ordonez, Weiqiang Zhou, Ajay Vaghasia, Anne Hamacher-Brady, Nathan R. Brady, Im-Hong Sun, Rulin Wang, Avi Z. Rosenberg, Michael Delannoy, Richard Rothman, Katherine Fenstermacher, Lauren Sauer, Kathyrn Shaw-Saliba, Evan M. Bloch, Andrew D. Redd, Aaron A.R. Tobian, Maureen Horton, Kellie Smith, Andrew Pekosz, Franco R. D'Alessio, Srinivasan Yegnasubramanian, Hongkai Ji, Andrea L. Cox, and Jonathan D. Powell

Table S1. Flow cytometry panels and antibodies used, related to Figures 1, 2, 5 and 6.

T cell Immuno-metabolic Panel

	MARKER	FLUOROPHORE	VENDOR	CAT #	CLONE
**SURFACE**	CD3	BV786	BD Biosciences	563800	SK7
	CD8	BV480	BD Biosciences	566121	RPA-T8
	CD45RA	BV570	Biolegend	304132	HI100
	CCR7	BV650	Biolegend	353234	G043H7
	CD25	BV510	BD Biosciences	563352	M-A251
	HLA-DR	BV750	Biolegend	307672	L243
	CXCR3	BV605	Biolegend	353728	G025H7
	PD-1	BV711	Biolegend	329928	EH12.2H7
	CD4	PE-Cy5	Biolegend	317412	OKT4
	CD69	PE-Cy5.5	ThermoFisher Scientific	MHCD6918	CH/4
	KLRG1	PE-CF594	BD Biosciences	565393	2F1
	CD49a	APC	Biolegend	328314	TS2/7
	CD19	APC-Cy7	Biolegend	363010	SJ25C1
	CD56	APC-Cy7	Biolegend	362512	5.1H11
**ICS**	FoxP3	PacBlue	Biolegend	320116	206D
	Tomm20	AF405	Abcam	ab210047	EPR15581-54
	VDAC1	AF532	Abcam	ab14734	20B12AF2
	CPT1a	AF488	Abcam	ab171449	8F6AE9
	Ki67	PE-Cy7	Biolegend	350526	Ki-67
	H3K27me3	PE	CST	40724	C36B11
	HK2	AF680	Abcam	ab228819	EPR20839
	GLUT1	AF647	Abcam	ab195020	EPR3915

B cell/Myeloid Immuno-metabolic Panel

	MARKER	FLUOROPHORE	VENDOR	CAT #	CLONE
**SURFACE**	CD14	BV605	Biolegend	301834	M5E2
	CD16	BV785	Biolegend	302046	3G8
	CD33	BV570	Biolegend	303417	WM53
	CD11c	BV480	BD Biosciences	74392	B-ly6
	HLA-DR	BV750	Biolegend	307672	L243
	CCR2	BV510	Biolegend	357218	K036C2
	CD40	PacBlue	Biolegend	334320	5C3
	CD38	BV711	BD Biosciences	563965	HIT2
	CD86	BV650	Biolegend	305428	IT2.2
	IgD	BB790	BD Biosciences	Custom order	IA6-2
	CD27	PE-CF594	BD Biosciences	562297	M-T271
	CD21	PE-Cy5	BD Biosciences	551064	B-ly4
	CD138	PE-Cy5.5	Biolegend	356502	MI15
	CD15	PE-Cy7	Biolegend	301924	HI98
	LOX1	PE	Biolegend	358604	15C4
	CD123	APC	Biolegend	306012	6H6
	CD3	APC-Cy7	Biolegend	344818	SK7
	CD19	APC-Cy7	Biolegend	363010	SJ25C1
	CD56	APC-Cy7	Biolegend	362512	5.1H11
**ICS**	Tomm20	AF405	Abcam	ab210047	EPR15581-54
	VDAC1	AF532	Abcam	ab14734	20B12AF2
	CPT1a	AF488	Abcam	ab171449	8F6AE9
	HK2	AF680	Abcam	ab228819	EPR20839
	GLUT1	AF647	Abcam	ab195020	EPR3915

Table S2. Characteristics of the 38 subjects with acute COVID-19, related to Figures 1-7.

**<u>Demographics</u>**	
Male N (%)	19 (50)
Female N (%)	19 (50)
Mean age (range)	59.7 (20-82)
Mean BMI (range)	32.2 (17.5-51.2)
Current smoker N (%)	0 (0)
**<u>Race and Ethnicity</u>**	
**Race**	N (%)
Black	17 (47.5)
White	11 (28.9)
Other*	7 (18.4)
Asian	2 (5.2)
**Ethnicity**	
Hispanic/Latinx	N (%)
Yes	5 (13.2)
No	32 (86.8)
**<u>Maximum Disease Severity</u>****	N (%)
$MinO_2$	14 (36.7)
HFO_2	4 (10.5)
Ventilated Lived	15 (39.5)
Died	5 (13.2)
**<u>Comorbidities</u>**	N (%)
Hypertension	21 (55.3)
Diabetes mellitus	15 (39.5)
COPD/asthma	12 (25.0)
Coronary artery disease	2 (5.2)
HIV infection	3 (7.9)

*Most self-identified as Hispanic/Latinx.

**Maximum disease severity indicates the most severe COVID-19 disease class for the patient while under observation: $MinO_2$= no or low flow oxygen required, HFO_2= high flow oxygen required, Ventilated= patient required intubation and survived, Died = patient died (ventilated or not)

Table S3. Characteristics of study subjects. Related to Figures 1-7.

	COVID-A	Influenza	COVID-R	Acute HCV	Chronic HCV
**Demographics**					
Male N (%)	19 (50)	9 (43)	6 (60)	2 (33)	7 (70)
Female N (%)	19 (50)	12 (57)	4 (40)	4 (67)	3 (30)
Mean age (range)	59.7 (20-82)	46.4 (22-89)	47.8 (18-81)	25.8 (24-28)	30.5 (26-35)

Supplemental Figure 1

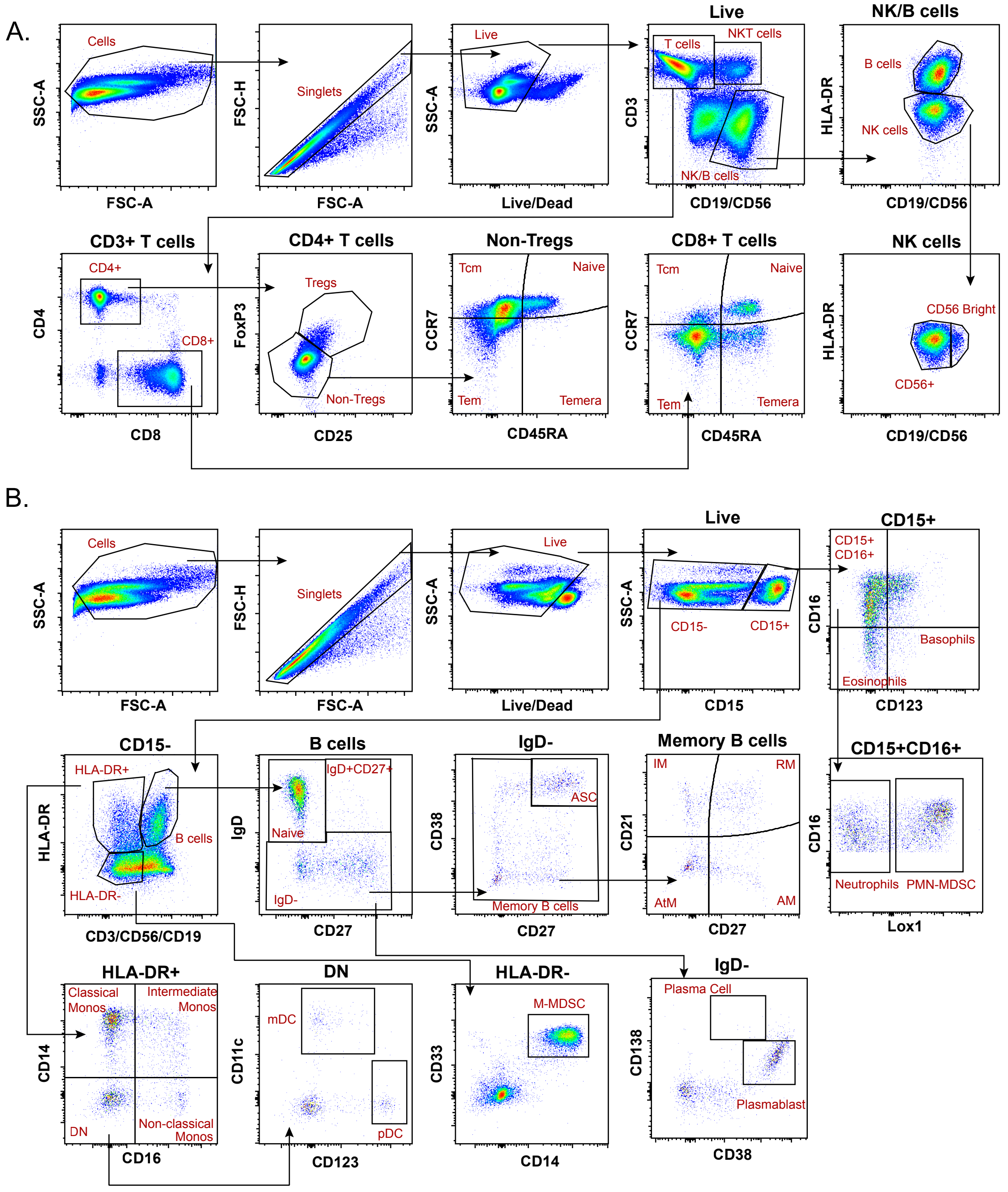

Fig. S1. Gating strategies for flow cytometry panels, related to Figures 1-2, 5-6. Representative flow plots from an acute COVID-19 subject show gating of all peripheral immune cell subsets assessed by one of two immuno-metabolic panels **(A)** T cell panel or **(B)** Myeloid and B cell panel.

Supplemental Figure 2

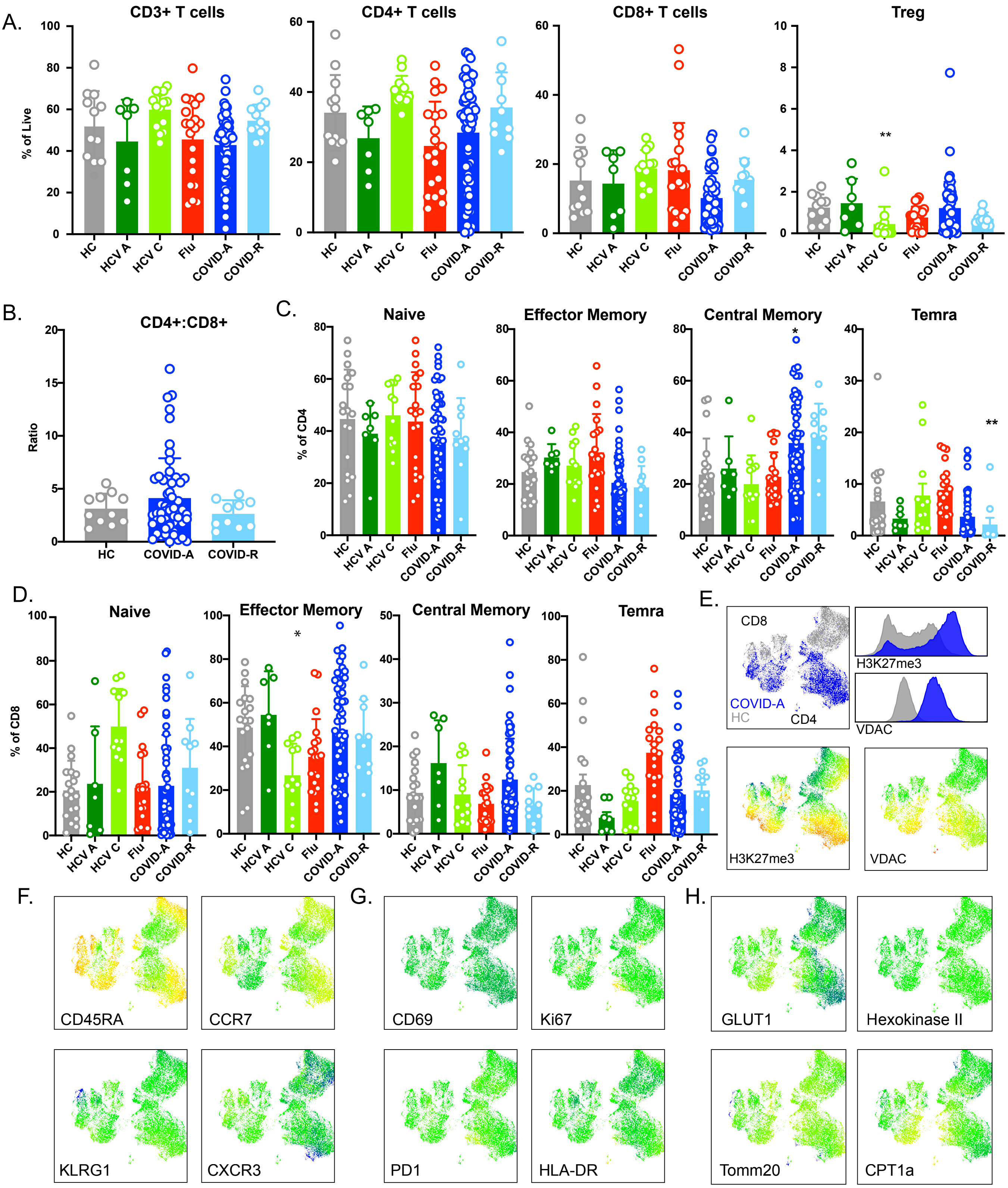

Fig. S2. Frequencies of T cell subsets and activation markers reveal few COVID19-specific differences, related to Figures 1 and 2.
(A) Frequency of indicated cell subset as percent of total live cells. Each dot represents one individual, significance tested using unpaired Kruskal-Wallis test compared to healthy control. **(B)** CD4:CD8 ratio. Each dot represents one individual, significance tested using unpaired Kruskal-Wallis test compared to healthy control. **(C)** Frequency of CD4+ and **(D)** CD8+ T cell subsets shown as percent of CD4 or CD8, respectively. Each dot represents one individual, significance tested using unpaired Kruskal-Wallis test compared to healthy control. **(E)** UMAP projection performed on a subset of COVID-A (blue) and HC subjects (grey). The two markers discovered to drive segregation of the COVID-A and HC cluster, H3K27Me3 and VDAC, are depicted as histogram overlays and MFI heatmap overlays on UMAP projection. **(F-H)** UMAP projection of MFI heatmap overlays of indicated proteins. Signifance is inidicated as compared to healthy control, *p<0.05, **p<0.01, ***p<0.001, ****p<0.0001, if no significance is indicated the test is non-significant.

Supplemental Figure 3

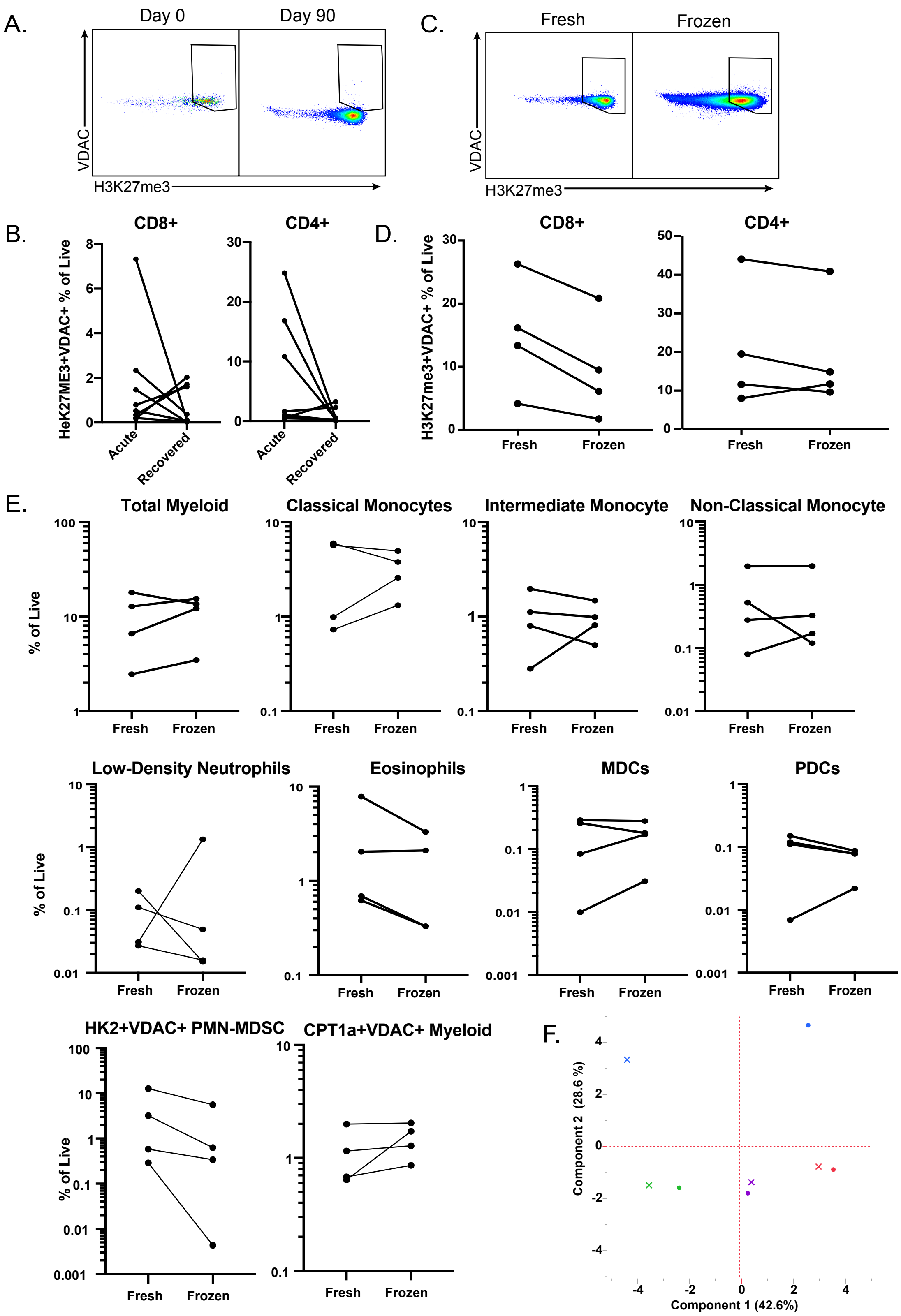

Fig. S3. Effect of cryopreservation and/or recovery on innate and adaptive compartments, related to Figures 1-2, 5-6.

(A) Representative plots show increased H3K27me3+VDAC+ CD8+ T cells in a COVID-A subject at day 0 enrollment compared to day 90 in the same subject after recovery. (B) Frequency of H3K27me3+VDAC+ T cells from 8 total COVID-A subjects with samples available at day 0 enrollment and after recovery for CD8+ T cells (left) and CD4+ T cells (right). Significance tested using Wilcoxon matched-pairs signed rank test. (C) Representative plots show increased H3K27me3+VDAC+ CD4+ T cells in a COVID-A subject with cells stained fresh compared to after cryopreservation. (D) Frequency of H3K27me3+VDAC+ T cells from 4 total COVID-A subjects tested at day 0 enrollment stained fresh or after cryopreservation for CD8+ (left) and CD4+ (right) T cells. Significance tested using Wilcoxon matched-pairs signed rank test. (E) Frequency of indicated cell subset as percent of total live cells when sample was processed fresh or after cryopreservation (frozen). Each dot represents one individual. Significance tested using Wilcoxon matched-pairs signed rank test. (F) Principal component analysis of frequencies of innate cell subsets show differences driven by patient variability and not cryopreservation.

Supplemental Figure 4

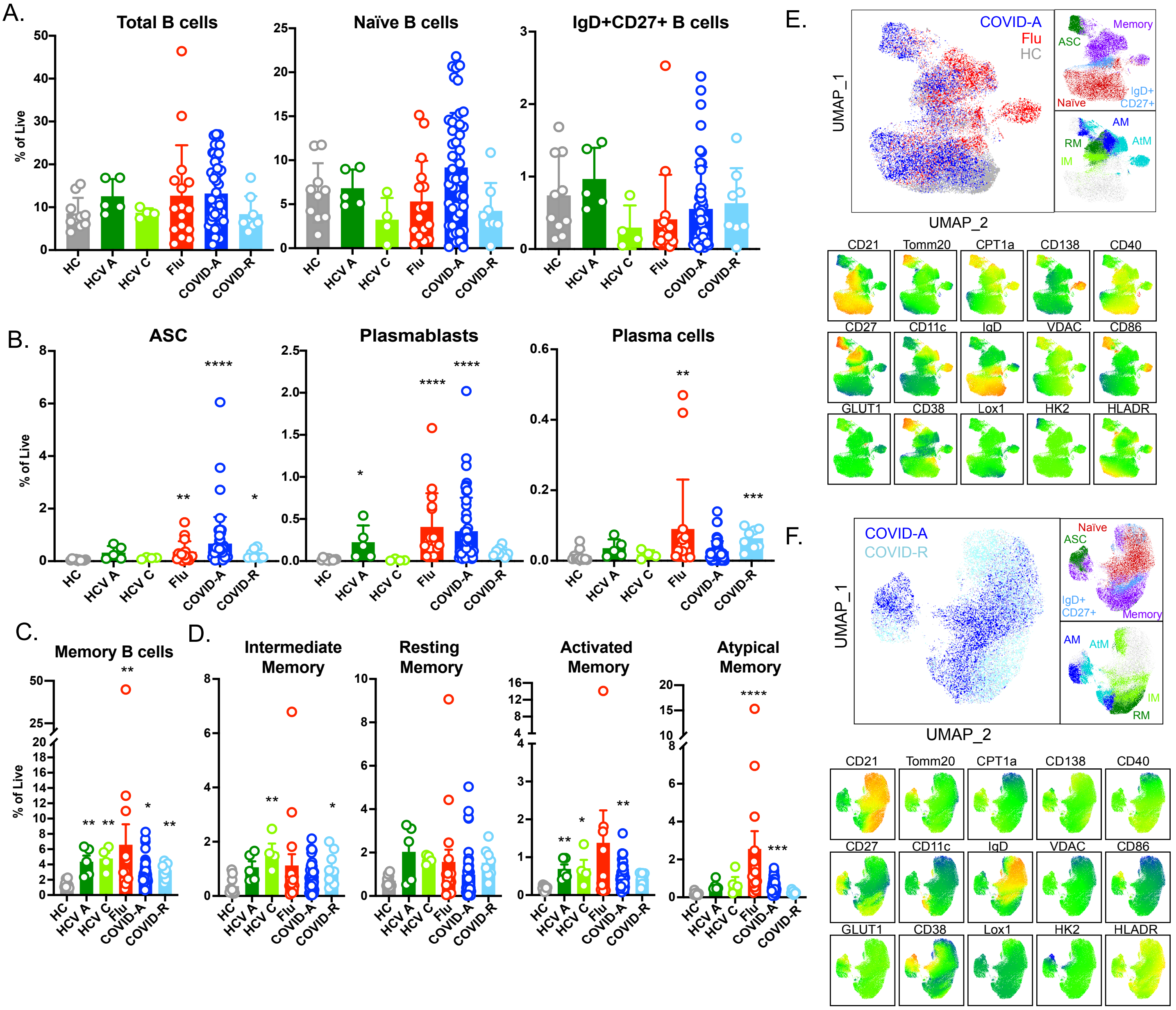

Fig. S4. B cell frequencies and phenotypes differ in the memory compartment in COVID-19, related to Figures 1 and 2.
(A-D) Frequency of indicated cell subset as percent of total live cells. Each dot represents one individual, significance tested using unpaired Kruskal-Wallis test compared to healthy control. **(E-F)** UMAP projection performed on a subset of COVID-A (blue), hospitalized Flu (red) and HC subjects (grey) **(E)** or COVID-A (blue) and COVID-R (light blue) **(F)**. Manual gating overlays on UMAP projection color code total B cell (top) and memory B cell (bottom) subsets. UMAP projection MFI heat maps of indicated proteins. Signifance is inidicated as compared to healthy control, *p<0.05, **p<0.01, ***p<0.001, ****p<0.0001, if no significance is indicated the test is non-significant.

Supplemental Figure 5

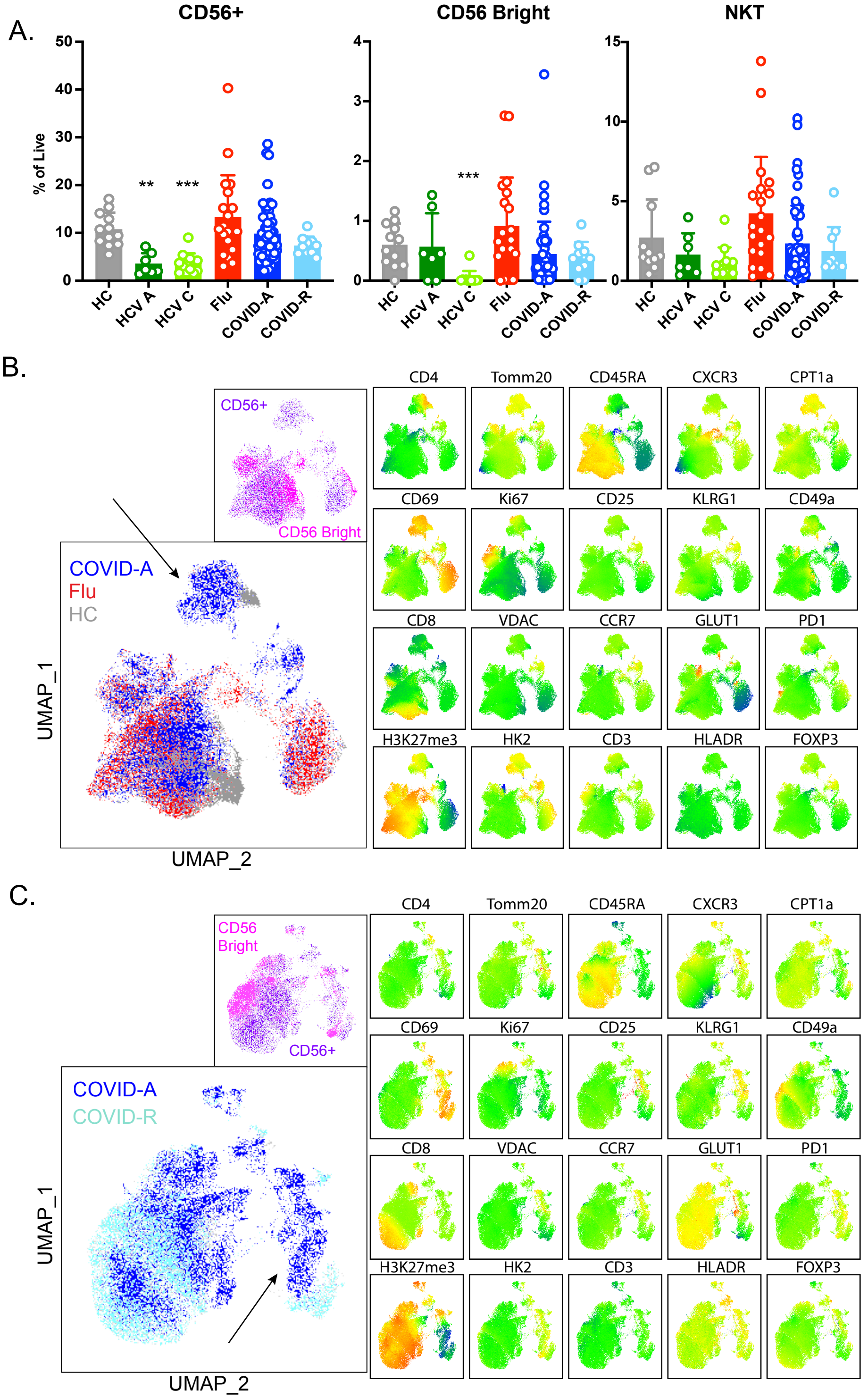

Fig. S5. Unique NK cell population in COVID-A subjects identified by high dimensional phenotyping analysis, related to Figures 1 and 2.
(A) Frequency of indicated cell subset as percent of total live cells. Each dot represents one individual, significance tested using unpaired Kruskal-Wallis test compared to healthy control. **(B)** UMAP projection of total NK cells performed on a subset of COVID-A (blue), hospitalized Flu (red) and HC subjects (grey) (left). Arrow indicates unique COVID-A specific cluster identified. Manual gating overlays on UMAP projection (top) color code CD56+ (purple) and CD56 bright (pink) cells. UMAP projection MFI heat maps of indicated proteins are shown right. **(C)** Similar analysis as in **(B)** was performed on a subset of COVID-A (blue) compared to COVID-R (light blue) subjects. Signifance is inidicated as compared to healthy control, *p<0.05, **p<0.01, ***p<0.001, ****p<0.0001, if no significance is indicated the test is non-significant.

Supplemental Figure 6

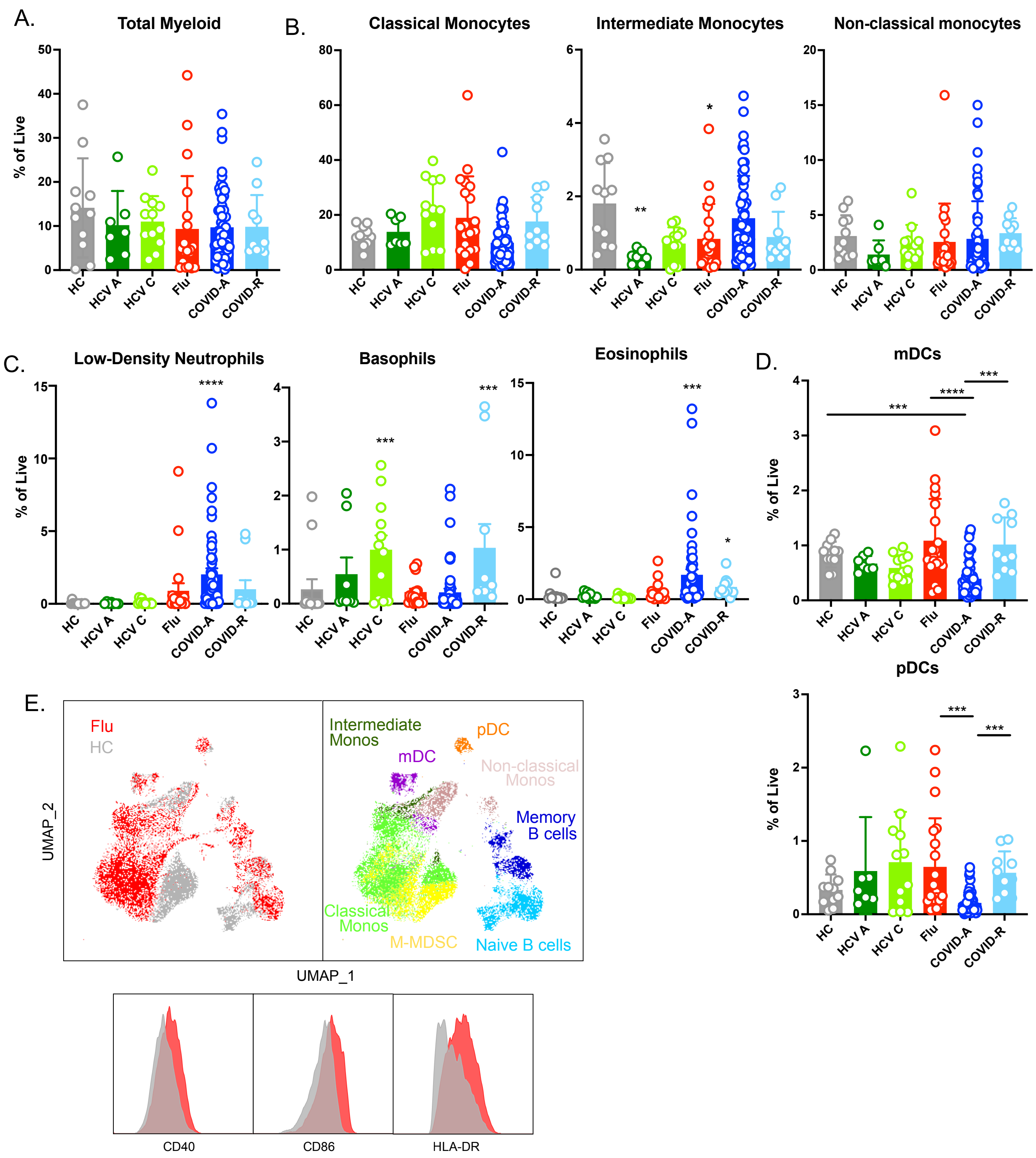

Fig. S6. Myeloid subsets in viral infections, related to Figures 5 and 6.
(A-C) Frequency of indicated cell subset as percent of total live cells. Each dot represents one individual, significance tested using unpaired Kruskal-Wallis test compared to healthy control. **(D)** Frequency of indicated cell subset as percent of total live cells. To asses how dendritic cell frequencies changed in recovery, significance was tested using unpaired Kruskal-Wallis test comparing all possible combinations. **(E)** UMAP projection of total myeloid cells performed on a subset of hospitalized Flu (red) and HC subjects (grey). Manual gating overlays on UMAP projection color code myeloid and B cell subsets in the UMAP space. MFI histogram overlays of CD14+ myeloid populations of indicated proteins for hospitalized Flu (red) and HC (grey). Signifance is inidicated as compared to healthy control (**A-C**), or between groups (**D**), *p<0.05, **p<0.01, ***p<0.001, ****p<0.0001, if no significance is indicated the test is non-significant.

Supplemental Figure 7

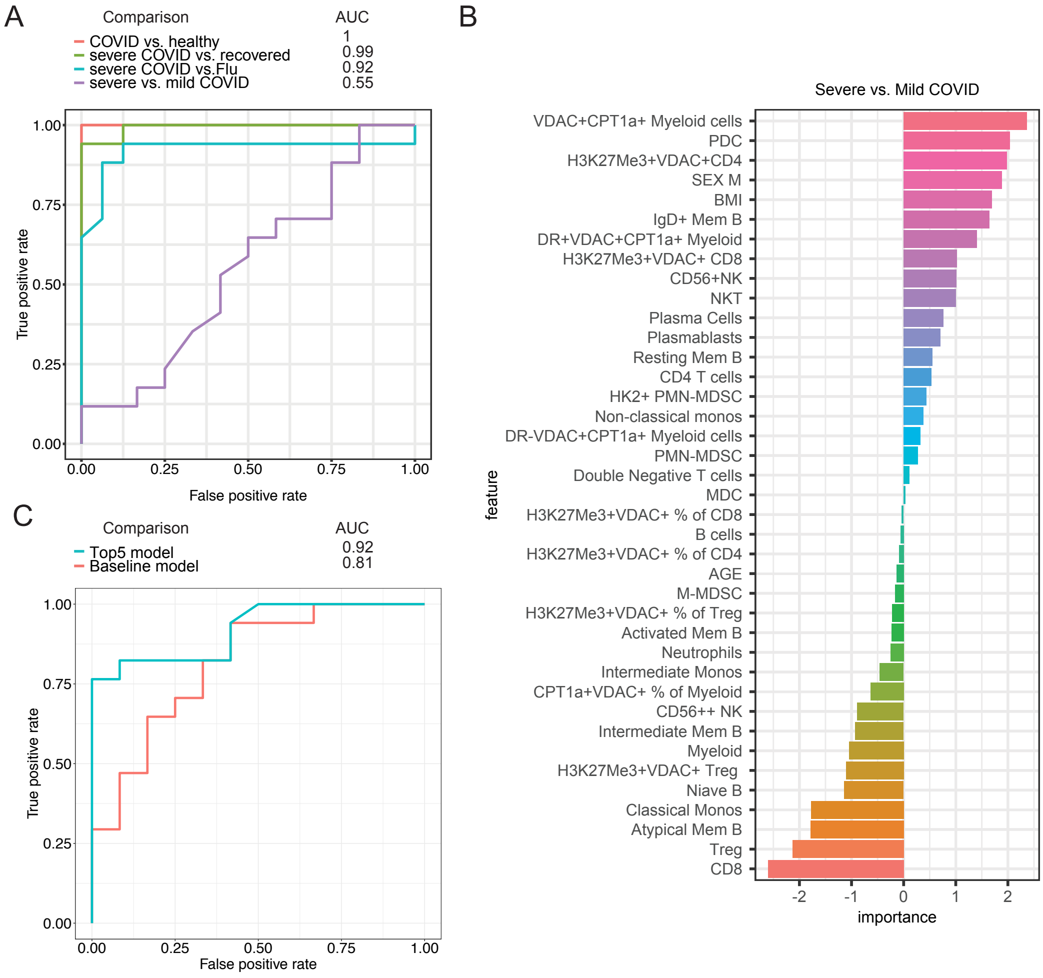

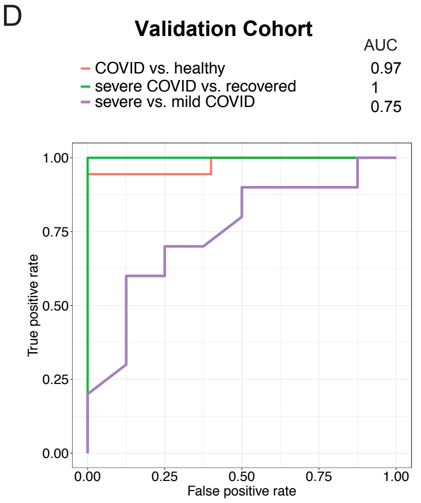

	mild vs severe COVID	severe COVID vs recovered	COVID vs HC
Accuracy	0.6667	1	0.7826
Sensitivity	0.5	1	0.7222
Specificity	0.875	1	1

Fig. S7. Metabolic profile of immune cells predicts disease status, related to Figure 7. **(A)** Receiver operating characteristic (ROC) curves for predicting different groups of patients (i.e., COVID-A vs. Healthy controls, Severe COVID-A vs. COVID-R, Severe COVID-A vs. Flu, and Severe vs. Mild COVID-A). The area under the curve (AUC) is indicated. **(B)** Feature importance analysis after adding basic clinical information (i.e., age, sex, and BMI) to the RF model for classifying severe vs. mild COVID-A. **(C)** ROC curves for comparing the performance of prediction models trained using the top-five-ranked features (i.e., top5) and basic clinical information (i.e., baseline) for classifying severe vs. mild COVID-A. AUC is indicated. **D.** The data were confirmed using a validation cohort including 5 COVID-R, 10 severe COVID-A, 8 mild COVID-A and 5 healthy controls (HC).
