# Supplementary material for: Metabolic programs define dysfunctional immune responses in severe COVID-19 patients
Source: Cell Rep. 2021 Feb 26;34(11):108863. doi: 10.1016/j.celrep.2021.108863 (PMC7908880; doi:10.1016/j.celrep.2021.108863)
Supplement: Document S2. Article plus ntal information [file mmc2.pdf]

Cell Reports

Article

Metabolic programs define dysfunctional immune responses in severe COVID-19 patients

Graphical abstract

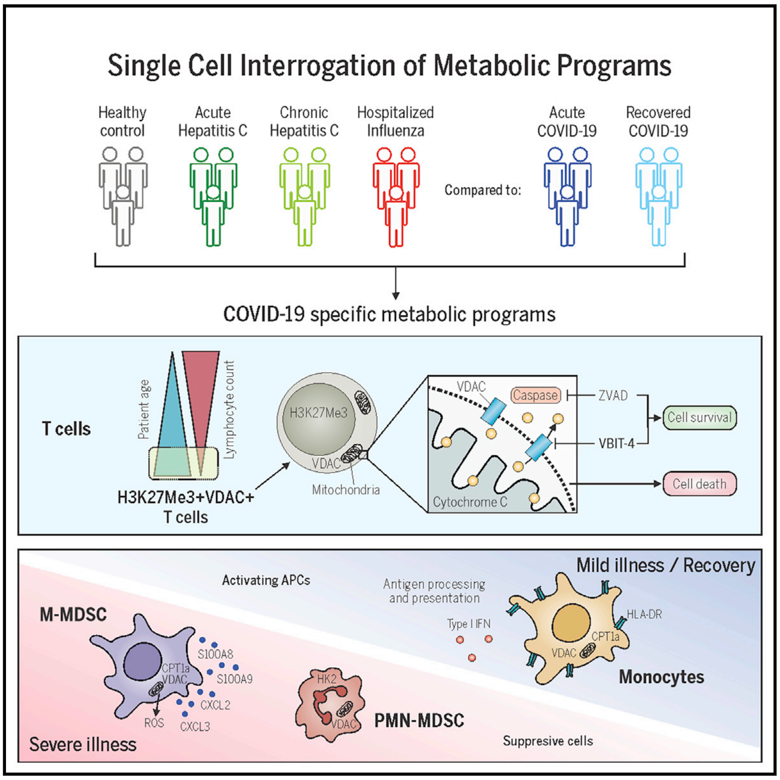

Highlights

- T cells with a unique metabolic profile are expanded in acute COVID-19
- These T cells are prone to mitochondrial apoptosis, correlating with lymphopenia
- Metabolically distinct myeloid-derived suppressor cells increase in acute COVID-19
- The presence of these M-MDSCs in acute COVID-19 correlates with disease severity

Authors

Elizabeth A. Thompson,
Katherine Cascino, Alvaro A. Ordonez, ...,
Hongkai Ji, Andrea L. Cox,
Jonathan D. Powell

Correspondence

acox@jhmi.edu (A.L.C.),
jpowell@jhmi.edu (J.D.P.)

In brief

The precise immunological defects that correlate with disease severity in COVID-19 have yet to be determined. Based on immune-metabolic profiling, Thompson et al. identify unique populations of T cells and myeloid cells that correlate with disease severity. These findings highlight metabolic pathways as possible therapeutic targets for COVID-19.

Thompson et al., 2021, Cell Reports *34*, 108863
March 16, 2021 © 2021 The Authors.
https://doi.org/10.1016/j.celrep.2021.108863

Cell Reports

CellPress
OPEN ACCESS

Article

Metabolic programs define dysfunctional immune responses in severe COVID-19 patients

Elizabeth A. Thompson,[1,2,11] Katherine Cascino,[3,11] Alvaro A. Ordonez,[4] Weiqiang Zhou,[5] Ajay Vaghasia,[1] Anne Hamacher-Brady,[6] Nathan R. Brady,[6] Im-Hong Sun,[1,2] Rulin Wang,[1] Avi Z. Rosenberg,[7] Michael Delannoy,[8] Richard Rothman,[9] Katherine Fenstermacher,[9] Lauren Sauer,[9] Kathyrn Shaw-Saliba,[9] Evan M. Bloch,[7] Andrew D. Redd,[3,10] Aaron A.R. Tobian,[7] Maureen Horton,[3] Kellie Smith,[1,2] Andrew Pekosz,[6] Franco R. D'Alessio,[3] Srinivasan Yegnasubramanian,[1,2,7] Hongkai Ji,[5] Andrea L. Cox,[1,2,3,6,*] and Jonathan D. Powell[1,2,12,*]

[1]Department of Oncology, Johns Hopkins University School of Medicine, Baltimore, MD 21287, USA
[2]Bloomberg~Kimmel Institute for Cancer Immunotherapy, Johns Hopkins University School of Medicine, Baltimore, MD 21287, USA
[3]Department of Medicine, Johns Hopkins University School of Medicine, Baltimore, MD 21287, USA
[4]Department of Pediatrics, Johns Hopkins University School of Medicine, Baltimore, MD 21287, USA
[5]Department of Biostatistics, Johns Hopkins University Bloomberg School of Public Health, Baltimore, MD 21287, USA
[6]W. Harry Feinstone Department of Molecular Microbiology and Immunology, Johns Hopkins University Bloomberg School of Public Health, Baltimore, MD 21287, USA
[7]Department of Pathology, Johns Hopkins University School of Medicine, Baltimore, MD 21287, USA
[8]Department of Cell Biology, Johns Hopkins University School of Medicine, Baltimore, MD 21287, USA
[9]Department of Emergency Medicine, Johns Hopkins University School of Medicine, Baltimore, MD 21287, USA
[10]Division of Intramural Research, National Institute of Allergy and Infectious Diseases, NIH, Baltimore, MD 21205, USA
[11]These authors contributed equally
[12]Lead contact
*Correspondence: acox@jhmi.edu (A.L.C.), jpowell@jhmi.edu (J.D.P.)
https://doi.org/10.1016/j.celrep.2021.108863

SUMMARY

It is unclear why some SARS-CoV-2 patients readily resolve infection while others develop severe disease. By interrogating metabolic programs of immune cells in severe and recovered coronavirus disease 2019 (COVID-19) patients compared with other viral infections, we identify a unique population of T cells. These T cells express increased Voltage-Dependent Anion Channel 1 (VDAC1), accompanied by gene programs and functional characteristics linked to mitochondrial dysfunction and apoptosis. The percentage of these cells increases in elderly patients and correlates with lymphopenia. Importantly, T cell apoptosis is inhibited *in vitro* by targeting the oligomerization of VDAC1 or blocking caspase activity. We also observe an expansion of myeloid-derived suppressor cells with unique metabolic phenotypes specific to COVID-19, and their presence distinguishes severe from mild disease. Overall, the identification of these metabolic phenotypes provides insight into the dysfunctional immune response in acutely ill COVID-19 patients and provides a means to predict and track disease severity and/or design metabolic therapeutic regimens.

INTRODUCTION

SARS-CoV-2 is a coronavirus responsible for the coronavirus disease 2019 (COVID-19) pandemic, resulting in over 20 million cases worldwide. Although the vast majority of infected patients experience a self-limiting viral syndrome, others develop severe disease leading to pneumonia and acute respiratory distress syndrome (ARDS), accounting for over 1 million deaths worldwide (Dong et al., 2020). At this time, it is unclear why some patients readily resolve infection while others develop severe symptoms. Specifically, it remains to be determined if severe disease is associated with a failure to generate protective immunity, overly robust dysfunctional immune responses, or a combination of both.

To date, severe COVID-19 has been associated with multiple changes in peripheral immune profiles, including lymphopenia and increased pro-inflammatory cytokines (Huang et al., 2020). Recent studies have broadly assessed immune profiles in COVID-19, revealing alterations in both the lymphocyte and myeloid compartments. Preferential loss of $CD8^+$ T cells, increased plasmablasts, neutrophil expansion, decreased plasmacytoid dendritic cells (pDCs), and differential T cell activation have been observed (Kuri-Cervantes et al., 2020; Laing et al., 2020; Mathew et al., 2020; Wilk et al., 2020). Although notable, these observations are indicative of generalized inflammation and thus fail to distinguish specific host deficiencies in SARS-CoV-2 infection versus other viral infections or inflammatory states.

It has become increasingly clear that metabolic reprogramming is not just a consequence of immune activation, but rather plays a critical role in facilitating immune cell differentiation and function (Buck et al., 2017; Patel et al., 2019). For example, effector T cells are characterized by increased expression of molecules necessary to support glycolysis, while memory T cells upregulate expression of molecules involved in oxidative phosphorylation

This is an open access article under the CC BY license (http://creativecommons.org/licenses/by/4.0/).

and fatty acid oxidation (Buck et al., 2015). Exhausted T cells are characterized not just by the upregulation of inhibitory molecules, such as programmed cell death protein 1 (PD-1), and loss of polyfunctionality but also by mTOR signaling in the absence of productive glycolytic function and anabolic processes (Bengsch et al., 2016). Therefore, we hypothesized that interrogation of immunometabolic phenotypes in COVID-19 has the potential to transcend traditional immune cell phenotyping and provide novel insights into distinct functional subsets. To this end, we developed a flow-cytometry-based proteomic and epigenetic approach that enables the interrogation of metabolic programs at the single-cell level (Table S1; Figure S1). We focused on three main pathways to characterize the metabolic landscape of immune cells in COVID-19. First, the mitochondrial membrane proteins Voltage-Dependent Anion Channel 1 (VDAC1) and Tomm20 were used as surrogates for mitochondrial mass and utilization of oxidative phosphorylation. Second, to evaluate the presence of glycolytic machinery used for glycolysis, we evaluated the glucose transporter Glut1 and the rate-limiting enzyme of glycolysis, hexokinase II (HKII). Finally, the ability to utilize fatty acid oxidation was measured by the expression of carnitine palmitoyltransferase 1a (CPT1a). Expression of H3K27Me3 was included as a readout for histone methylation, which is regulated at multiple levels by metabolites of the TCA cycle (Pearce and Shen, 2006). Using this approach, we identified distinct T cell and myeloid subsets in the peripheral blood mononuclear cells (PBMCs) of acutely ill COVID-19 (COVID-A) patients who were not found in the PBMCs of recovered COVID-19 (COVID-R) patients and patients with hepatitis C and influenza viral infections. Furthermore, these cells provide important mechanistic insight into the immune dysfunction observed in COVID-A patients and the mechanism of pathogenesis.

RESULTS

Identification of a distinct T cell subset in the PBMCs of COVID-A patients

The high-dimensional flow-cytometry-based assay was performed on thawed PBMCs from an institutional review board (IRB)-approved biorepository from patients admitted to Johns Hopkins Hospital (Table S2). We initially focused on T cells within the PBMCs given their importance in viral control. Using traditional immunological markers, we observed limited differences in T cell frequencies and phenotype, as previously described (Mathew et al., 2020; Mazzoni et al., 2020). Most notably, we detected an increase in the CD4/CD8 ratio and an increase in central memory CD4 T (Tcm) cells in COVID-A patients when compared with healthy controls (Figure S2).

In stark contrast, unbiased analysis of T cells employing the combined immune and metabolic markers dramatically segregated T cells from healthy controls and COVID-A (Figure 1A). When compared with healthy controls, we observed no differences in expression of the classical markers of T cell subsets CD45RA and CCR7 (Figure S2F) or the activation markers CD69, Ki67, PD-1, or histocompatibility leukocyte antigen (HLA)-DR (Figure S2G). Likewise, levels of metabolic enzymes involved in glycolysis and fatty acid oxidation did not differ between the two groups (Figure S2H). In contrast, we identified a unique population of T cells found in the COVID-A patients characterized by robust upregulation of VDAC1 and the epigenetic marker H3K27me3 (Figures 1A and 1B). H3K27me3 is regulated in part by α-ketoglutarate-mediated jumonji demethylases (Pearce and Shen, 2006), while VDAC1 is a mitochondrial membrane protein involved in metabolite transport and has been associated with mitochondrial cell death signaling and lupus-like autoimmunity (Ben-Hail et al., 2016; Kim et al., 2019). This phenotype was unique in its exclusive upregulation of mitochondrial proteins, without a concomitant upregulation of glycolytic machinery. Classically, upon T cell activation, VDAC1 and H3K27me3 expression increase along with the glucose transporter Glut1 and HKII, both of which support activation-induced glycolysis. This traditional activation signature was seen upon TCR stimulation of healthy PBMCs *in vitro* (Figure 1C). However, the unique population of H3K27me3^{hi}VDAC1^{hi} T cells in the PBMCs of COVID-A patients differ from conventional recently activated T cells because they express relatively low levels of Glut1 and HKII (Figure 1D). The H3K27me3^{hi}VDAC1^{hi} T cells were found within both the naive and the memory T cell compartment (Figure 1D) and did not demonstrate enrichment for specific TCR clones (Figure 1E). Expanded analysis of 55 PBMC samples from 38 COVID-A patients (including sequential samples) revealed that these T cells were markedly upregulated (many exceeding 50% of total T cells) in COVID-A patients and were almost completely absent in healthy controls (Figure 1F). Interestingly, although not present in all of the patients queried, every COVID-A patient 70 years of age and older demonstrated frequencies of the H3K27me3^{hi}VDAC1^{hi} T cells that were 40% or greater in their PBMCs (Figure 1G). Overall, these data reveal a distinct population of H3K27me3^{hi}VDAC1^{hi} T cells in the PBMCs of COVID-A patients. The discordance in activation and metabolic phenotypes suggests that these T cells are dysfunctional.

The unique population of H3K27me3^{hi}VDAC1^{hi} T cells distinguishes COVID-A patients from recovered patients and hospitalized patients infected with influenza

By interrogating metabolic programs using our immune-metabolic assay, we were able to identify a unique population of H3K27me3^{hi}VDAC1^{hi} T cells in COVID-A patients. We next wanted to determine if this population of T cells was unique to the COVID-A patients. To this end, we interrogated the PBMCs of COVID-R patients (>28 days from diagnosis) and patients with other viral infections to determine if this phenotype was simply a result of ongoing inflammation or a general consequence of viral infection. PBMCs were obtained and analyzed from patients with acute and chronic viral hepatitis C infection (Cox et al., 2005), patients hospitalized with influenza infection (Dugas et al., 2020), and COVID-R patients (Klein et al., 2020) (Table S3). There appeared to be only subtle differences between these groups in CD4^{+} and CD8^{+} T cell subsets as defined by traditional markers (Figure S2). Yet, when including metabolic markers in the analysis, the COVID-A patients' T cells again segregated compared with other groups as shown by UMAP projections, indicating a distinct subset of T cells unique to COVID-A patients not accounted for by traditional markers (Figures 2A–2C). H3K27me3^{hi}VDAC1^{hi} T cells were absent in both acute and chronic hepatitis C infection; however, they were present in a

CellPress
OPEN ACCESS

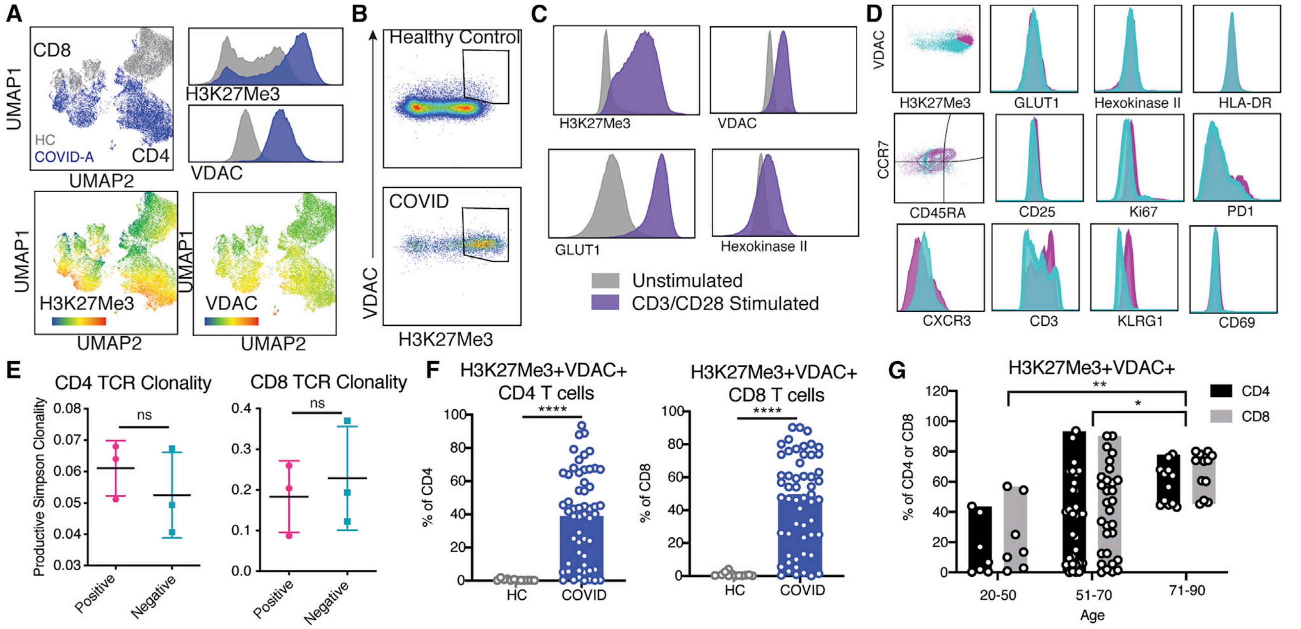

Figure 1. Identification of novel metabolically distinct T cells in COVID-19 patients

(A) Concatenated flow cytometry data depicted as UMAP projection of $CD3^+$ T cells from healthy control (HC, gray) and hospitalized acute COVID-19 (COVID-A) patients (blue). The two markers discovered to drive segregation of the COVID-A and HC cluster, H3K27Me3 and VDAC1, are depicted as histogram overlays and MFI heatmap overlays on UMAP projection.

(B) Representative flow plots of $H3K27Me3^+VDAC1^+$ T cells.

(C) Healthy PBMCs were stimulated for 24 h with anti-CD3 and anti-CD28 and evaluated for metabolic enzymes using flow cytometry. Representative histograms of unstimulated (gray) and stimulated (purple) cultures.

(D) Histograms comparing $H3K27Me3^+VDAC1^+$ T cells (pink) and remaining T cells (blue) from concatenated pooled COVID-A donors for indicated proteins.

(E) TCR Simpson clonality from sorted $H3K27Me3^+VDAC1^+$ T cells (pink) and remaining T cells (blue).

(F) Frequency of $H3K27Me3^+VDAC1^+$ as percent of CD4 or CD8 T cells. Each dot represents one patient sample; significance was tested using unpaired Mann-Whitney test.

(G) Frequency of $H3K27Me3^+VDAC1^+$ as percent of CD4 (black) or CD8 (gray) stratified by age of COVID-A patients.

*$p < 0.05$, **$p < 0.01$, ***$p < 0.001$, and ****$p < 0.0001$.

subset of both influenza and COVID-R patients (Figures 2D and 2E). Longitudinal samples were available for eight COVID-A patients, three of which had elevated levels of $H3K27me3^{hi}VDAC1^{hi}$ T cells at baseline. In these patients, there was a marked reduction in the percentage of these T cells at day 90 during recovery (Figures S3A and S3B).

Importantly, although T cells present in severe influenza infection and during recovery maintained elevated VDAC1 and H3K27me3 expression, clustering the $H3K27me3^{hi}VDAC1^{hi}$ CD4 T cells by protein expression of all other markers included in the flow cytometry panel showed that these cells robustly clustered based on disease type, indicating qualitative differences in $H3K27me3^{hi}VDAC1^{hi}$ T cells across disease status (Figure 2F). That is, even though T cells expressing H3K27me3 and VDAC1 were present in the PBMCs of the influenza and COVID-R patients, they were distinct from the $H3K27me3^{hi}VDAC1^{hi}$ T cells in the PBMCs of the COVID-A patients.

For example, as described earlier, the $H3K27me3^{hi}VDAC1^{hi}$ T cells from the COVID-A patients demonstrated significantly decreased expression of Glut1, a metabolic marker associated with T cell effector function, compared with COVID-R and influenza (Figure 2G). Notably, the level of Glut1 on the $H3K27me3^{hi}VDAC1^{hi}$ T cells corresponded to disease severity in COVID-A patients. Although the most severe patients (requiring mechanical ventilation) expressed significantly lower levels of Glut1, the mild patients (hospitalized and requiring low or high flow oxygen) demonstrated $H3K27me3^{hi}VDAC1^{hi}$ T cells with Glut1 levels similar to COVID-R patients and influenza patients (Figure 2G). In contrast, $H3K27me3^{hi}VDAC1^{hi}$ T cells from COVID-A patients had significantly increased expression of the mitochondrial protein Tomm20 and KLRG1, a marker associated with T cell senescence and age-related functional defects (Henson and Akbar, 2009) (Figure 2G). Thus, despite similar levels of H3K27Me3 and VDAC1 expression, the expression of proteins involved in metabolic programming of these cells is distinct between severe COVID-A patients and mild COVID-19/COVID-R or hospitalized influenza patients, all of whom recovered. Therefore, these cells represent a population truly unique to COVID-A and may provide insight into the immune dysregulation observed in SARS-CoV-2 infection.

Dysfunctional mitochondria in the T cells of COVID-A patients leads to apoptosis in a VDAC1-dependent fashion

In order to elucidate the functional significance of these T cells that were highly prevalent in COVID-A, we performed single-cell RNA sequencing (RNA-seq) on six COVID-A patients, all of

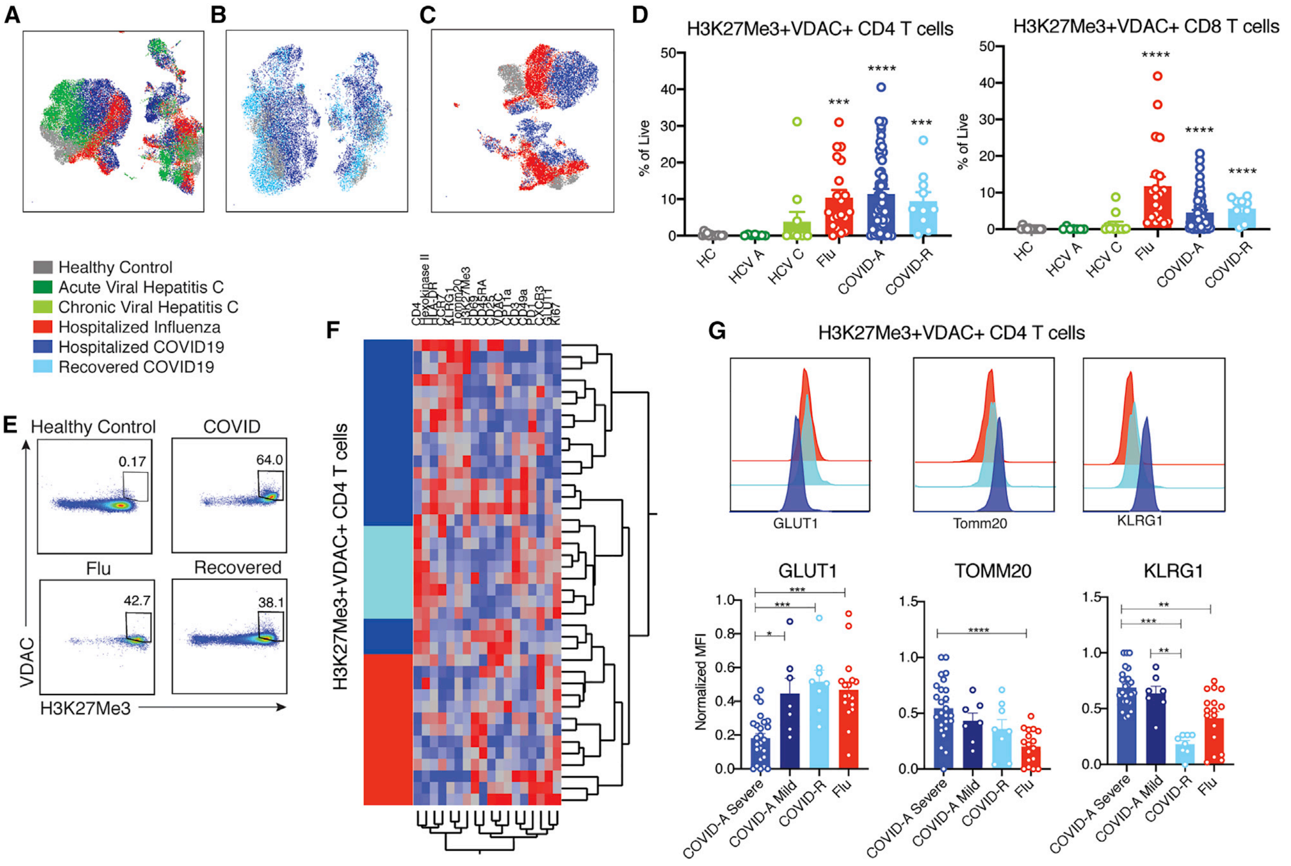

Figure 2. Novel H3K27me3$^+$VDAC1$^+$ T cells are unique in COVID-19 compared with other viral infections

(A) UMAP projection of pooled donors with active infection, color coded by disease.

(B) UMAP projection of COVID-A and recovered COVID-19 (COVID-R) patients compared with HCs.

(C) UMAP projection of influenza and COVID-A patients compared with HCs.

(D) Frequency of H3K27Me3$^+$VDAC1$^+$ CD4 and CD8 T cells as percent of total live cells.

(E) Representative gating of H3K27Me3$^+$VDAC1$^+$ CD4 T cells from multiple disease states.

(F) Hierarchical clustering of H3K27Me3$^+$VDAC1$^+$ CD4 T cells based on expression (MFI values) of indicated proteins. Comparison of hospitalized COVID-A infection (dark blue), COVID-R (light blue), and hospitalized influenza (red).

(G) Normalized MFI of GLUT1, TOMM20, and KLRG1 on H3K27Me3$^+$VDAC1$^+$ CD4 T cells in all patients where H3K27Me3$^+$VDAC$^+$ T cells compromise greater than 10% of the total CD4 population and representative histogram overlays of MFI.

Each dot represents one patient sample; significance was tested using unpaired Kruskal-Wallis test compared with HC (D) or every combination (G). *$p < 0.05$, **$p < 0.01$, ***$p < 0.001$, ****$p < 0.0001$.

which had more than 50% of the T cells of interest as determined by flow cytometry, and three healthy controls. Evaluating gene programs enriched in T cells derived from COVID-A patients revealed an upregulation of cell death programs, as has been reported previously (Zhang et al., 2020) (Figure 3A, red bars). Furthermore, these data demonstrated evidence of mitochondrial dysfunction, including downregulation of several programs associated with mitochondria function, organization, respiratory chain complex assembly, oxidative phosphorylation, and electron transport-coupled proton transport (Figure 3A, blue bars).

To investigate whether elevated levels of VDAC1 and Tomm20 were indicative of altered mitochondrial function in these T cells, we performed electron microscopy (EM) on PBMCs from additional COVID-A patients and healthy controls to examine the lymphocyte mitochondria. This analysis revealed markedly dysmorphic, irregularly shaped mitochondria with incomplete cristae in lymphocytes from COVID-A patients compared with lymphocytes from healthy controls, consistent with dysregulated mitochondrial function (Figure 3B). In addition to displaying morphological characteristics of apoptosis, lymphocytes from COVID-A patients showed prominent degenerative changes, including large cytoplasmic vacuoles.

We next performed confocal microscopy of PBMCs stained with anti-CD3 and MitoTracker Deep Red, which stains mitochondria. CD3$^+$ T cells from COVID-A patients demonstrated less distinct mitochondrial staining versus healthy controls (Figure 3C), consistent with the dysmorphic mitochondria observed by EM. Based on these findings and along with the observed decreased mitochondrial programs by RNA-seq and the presence of apoptotic cell morphologies by EM, we hypothesized

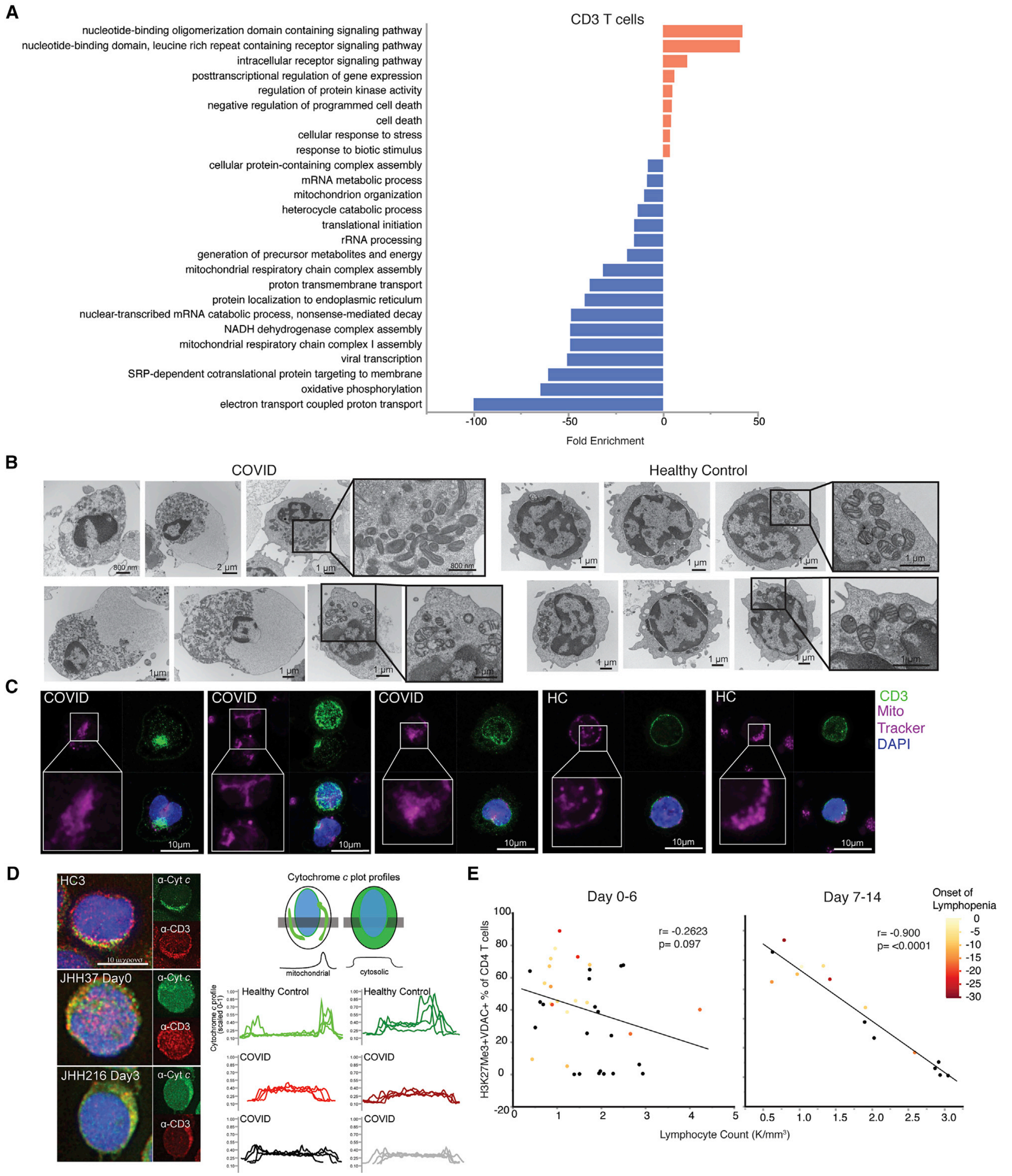

Figure 3. T cells from COVID-19 subjects demonstrate parameters of mitochondrial dysfunction and apoptosis signaling, correlating with development of lymphopenia

(A) Single-cell RNA sequencing analysis of six COVID-19 subjects and three HCs were evaluated for $CD3^+$ T cells. Genes distinguishing T cells from COVID-19 patients compared with HCs were evaluated for statistical overrepresentation using Gene Ontology (GO) biological processes as gene sets and categorized into

(legend continued on next page)

that VDAC1 might act to facilitate release of mitochondrial cytochrome *c* to the cytosol, leading to apoptosis (Camara et al., 2017; Garrido et al., 2006). We therefore performed immunofluorescence analysis of endogenous cytochrome *c* and found it to be present in the cytoplasm of a subset of CD3$^+$ T cells from COVID-A patients, whereas in healthy controls, cytochrome *c* was localized to the mitochondria (Figure 3D). Of note, although release of cytochrome *c* into the cytoplasm can lead to rapid cell death, in our procedure, the cells were fixed immediately after thawing, prior to measurement of endogenous cytochrome *c.* Employing this technique enabled us to capture a subset of T cells undergoing active mitochondria-induced apoptosis. Interestingly, the presence of H3K27me3$^+$VDAC1$^+$ T cells was highly predictive of the development of lymphopenia during the course of hospitalization. We observed a highly significant ($p < 0.0001$) negative correlation between the frequency of these cells and the peripheral lymphocyte count as measured after 7 days of hospitalization (Figure 3E). This highly significant negative correlation supports the hypothesis that mitochondrial-induced apoptosis is in part contributing to the lymphopenia that develops in the COVID-A patients during the course of their disease (Kuri-Cervantes et al., 2020; Mathew et al., 2020). Thus, the unique population of T cells found in the COVID-A patients display mitochondrial dysfunction consistent with cytochrome *c* release into the cytoplasm and subsequent induction of apoptosis.

Given these findings and the fact that VDAC1 can facilitate caspase-mediated cell death (Abu-Hamad et al., 2009; Camara et al., 2017; Shimizu et al., 2000), we hypothesized that the high expression of VDAC1 in the setting of dysmorphic mitochondria was directly linked to increased susceptibility to cell death in these T cells. To test this hypothesis, we cultured PBMCs from COVID-A patients and healthy controls for 48 h *in vitro* in the presence of media alone, the mTOR inhibitor rapamycin, the VDAC1 oligomerization inhibitor VBIT-4 (Ben-Hail et al., 2016; Kim et al., 2019), or the global caspase inhibitor ZVAD (Figure 4A). We observed decreased T cell survival in media alone from the PBMCs of COVID-A patients when compared with healthy controls, confirming increased cell death in COVID-A T cells. Interestingly, survival of the COVID-A T cells was rescued with either the VDAC1 oligomerization inhibitor or the pan-caspase inhibitor, but not the mTOR inhibitor. These findings are consistent with the fact that VDAC1 oligomerization and interaction with BCL2 family proteins is thought to enable pore formation within the mitochondrial outer membrane, allowing for cytoplasmic release of cytochrome *c*, which initiates the caspase cascade to induce cellular apoptosis (Abu-Hamad et al., 2009; Camara et al., 2017; Shimizu et al., 2000). Notably, neither VBIT-4 nor ZVAD enhanced survival of the healthy T cells, demonstrating that this mechanism of cell death contributes to loss of viability in the COVID-A T cells, but not in the T cells from the healthy controls. Interestingly, the COVID-A T cells treated with ZVAD and VBIT-4 treatment demonstrated a robust response to anti-CD3 + anti-CD28 stimulation, similar to the healthy T cells, indicating the ability to functionally rescue the T cells (Figure 4B). These data, combined with the observations of increased cytoplasmic cytochrome *c*, suggest that VDAC1 is promoting cell death through direct pore formation (Figure 4C). However, these observations do not rule out a role for an interaction between VDAC1 and BAX/BAK to promote cell death in these T cells.

In light of the propensity of the T cells from the COVID-A patients to undergo apoptosis, we wanted to verify that our results did not reflect differences in cell survival after freezing. When we compared fresh and frozen PBMCs from four COVID-A patients, we observed an increased frequency of the H3K27me3^{hi}VDAC1^{hi} T cells in the freshly stained PBMCs (Figures S3C and S3D) compared with those frozen and thawed. Thus, our studies are potentially underestimating the frequency of these unique T cells in the COVID-A patients.

Immune metabolic evaluation of B cells and NK cells

Using our assay, we also interrogated the metabolic programs of B cells and NK cells. In contrast with T lymphocytes, we did not observe marked differences in the frequencies of naive B cells between any viral infections and the healthy controls (Figure S4). Consistent with recent reports, we did observe an increase in the peripheral antibody-secreting cells (ASCs) in the COVID-A patients (Kuri-Cervantes et al., 2020). All of the viral infections were associated with a significant increase in the memory B cells versus healthy controls, while only the COVID-A and hepatitis C patient groups demonstrated an increase in the activated memory B cells. The COVID-A and influenza patient groups both had a higher percent of atypical memory B cells compared with PBMCs from the hepatitis C and the COVID-R patient groups. Overall, global UMAP projection demonstrated only subtle phenotypic differences among the different groups in the B cell compartment. When we examined the NK cells, we found no significant differences in frequency when comparing the COVID-A patients, the COVID-R patients, and the influenza patients (Figure S5). Conversely, HD flow analysis revealed a population of CD56$^+$ NK cells in the COVID-A patients not present in the influenza patients or in the healthy controls. This population was defined by the upregulation of classical activation markers CD69, Ki67, and CD49a and by increased expression of the metabolic markers Tomm20, CPT1a, and HKII. When we compared NK cells from COVID-A patients with COVID-R patients, we observed

higher-level annotation using ReviGO. Displayed is the enrichment score for each gene set, and color corresponds to programs in upregulated genes (red) and downregulated genes (blue).
(B) Representative electron microscopy images of PBMCs from a COVID-A patient and a HC.
(C) Representative confocal images of PBMCs from a COVID-A patient and HC with mitochondria labeled using MitoTracker Deep Red (pink) and CD3$^+$ T cells labeled (green) and nuclei labeled with DAPI (blue).
(D) Representative fluorescence images of PBMCs from three COVID-A subjects and one HC (left) immunostained for cytochrome *c* (green) and CD3 (red), and nuclei labeled with DAPI (blue). Plot profiles of intracellular cytochrome *c* fluorescence intensity distribution (right).
(E) Correlation of lymphocyte count with frequency of H3K27Me3$^+$VDAC1$^+$ out of total CD4$^+$ T cells from days 0–6 (left) or days 7–14 (right) of study enrollment, including all symptomatic patients. Each dot represents one subject, and color corresponds to days since onset of lymphopenia (black = no COVID-19-related lymphopenia developed). Correlation tested using non-parametric Spearman's correlation.

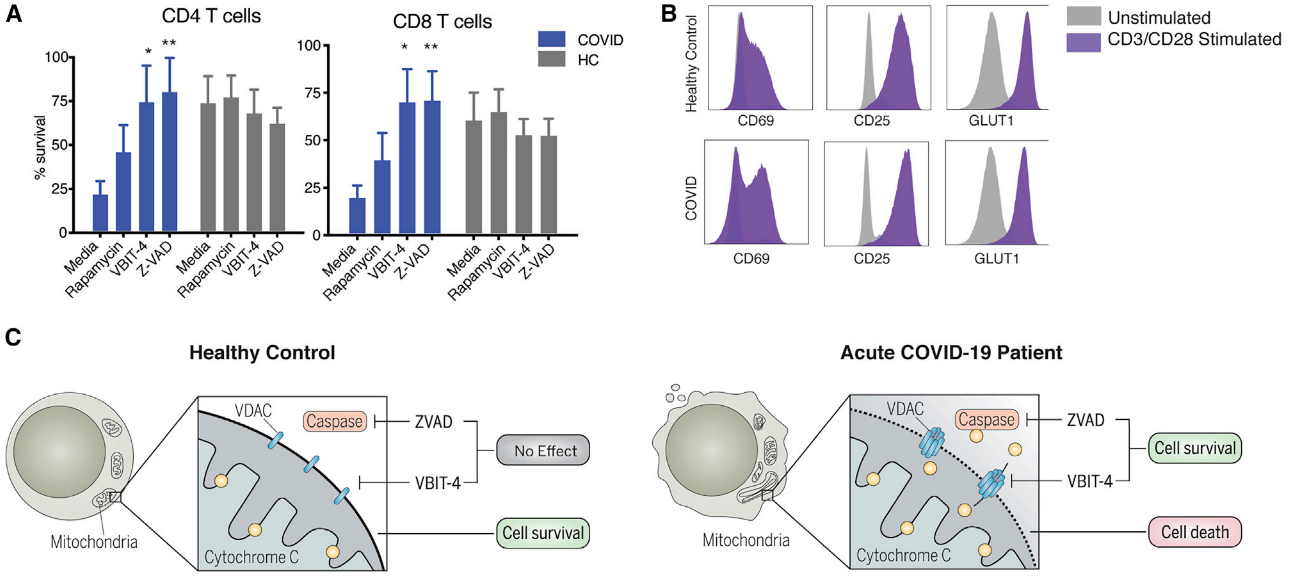

Figure 4. Loss of T cell survival can be rescued by targeting VDAC1 or caspases

(A) PBMCs from COVID-A patients or HCs were cultured for 48 h in media, rapamycin (100 nM), VBIT-4 (300 nM), or ZVAD (60 nM). T cell survival was calculated as the percent CD4 or CD8 T cells remaining from initial plating. Significance was tested using two-way ANOVA with each drug compared with the media control, n = 9.

(B) T cells were stimulated with anti-CD3/CD28 (purple) for 48 h, and surviving T cells from COVID-19 patients are able to respond by upregulating HLA-DR, CD69, CD25, and GLUT1 to the same extent as HCs compared with unstimulated controls (gray).

(C) Graphical depiction of proposed mechanism of mitochondrial cell death signaling in COVID-19 T cells.

*$p < 0.05$, **$p < 0.01$, ***$p < 0.001$, ****$p < 0.0001$.

differences in CD56^{+} and CD56^{bright} cells driven mainly by differential expression of markers of activation. Although the precise significance of these cells is unclear, their generation can potentially provide important clues into the dysregulated systemic inflammation characteristic of acutely ill COVID-A patients.

Identification of a unique population of VDAC1^{+} HKII^{+} polymorphonuclear myeloid-derived suppressor cells (PMN-MDSCs) in the PBMCs of COVID-A patients, but not recovered patients or patients with other viral illnesses

Next, we examined myeloid cells in the PBMCs of the COVID-A patients employing our HD immuno-metabolic assay (Figure S6). Consistent with prior reports, COVID-A was associated with a decreased percentage of myeloid dendritic cells (mDCs) and pDCs in PBMCs and limited differences in monocyte populations (Kuri-Cervantes et al., 2020; Laing et al., 2020) (Figures S6A–S6C). Although cryopreservation is known to affect the recovery of myeloid populations, a rapid protocol to stain cells immediately post-thaw was utilized to minimize this effect and allowed for recovery of monocytic and granulocytic cells post-cryopreservation, when compared with freshly isolated cells (Figures S3E and S3F).

Visualization of myeloid cells by UMAP projection revealed two distinct populations, which once again only became apparent upon interrogating metabolism (Figures 5A and 5B). We first identified CD15^{+} granulocytic cells in COVID-A patients, which were entirely absent from the healthy controls (Figures 5A and 5B). Evaluating the granulocytic cells further revealed a combination of low-density neutrophils, basophils, and PMN-MDSCs (Figure 5C; Figure S6). Increased neutrophil counts have been previously observed in COVID-19 patients (Kuri-Cervantes et al., 2020; Laing et al., 2020), and low-density neutrophils can be found in the PBMC fraction during inflammation, representing both immature neutrophils and activated/degranulated neutrophils (Silvestre-Roig et al., 2019). Immunosuppressive PMN-MDSC are often associated with chronic inflammation, as is seen in cancer, obesity, and chronic viral infection (Zhou et al., 2018). Although low levels of PMN-MDSCs were detectable in chronic hepatitis C, there were significantly more in COVID-A patients (Figure 5C). Of note, the PMN-MDSCs expressed the highest levels of VDAC1 within the PBMCs, higher than the H3K27Me3^{hi}VDAC1^{hi} pre-apoptotic T cells (Figure 5D). However, in contrast with the VDAC1^{+} T cells, the PMN-MDSCs had concurrent upregulation of HKII, which is known to associate with VDAC1 and prevent apoptosis (Shoshan-Barmatz et al., 2009). Thus, the granulocytic suppressor cells (PMN-MDSCs) in the PBMCs of the COVID-A patients possessed a unique metabolic signature (Figure 5E). The expression of VDAC1 and HKII provide further information into the metabolic programing of these cells and may provide potential therapeutic targets for an immunosuppressive MDSC compartment.

Identification of a unique population of CPT1a^{+} VDAC1^{+} DR^{-} monocytic-MDSCs (M-MDSCs) in the PBMCs of COVID-A patients that is associated with disease severity

We next identified a population of monocytic cells present in COVID-A patients who expressed high levels of CPT1a, an

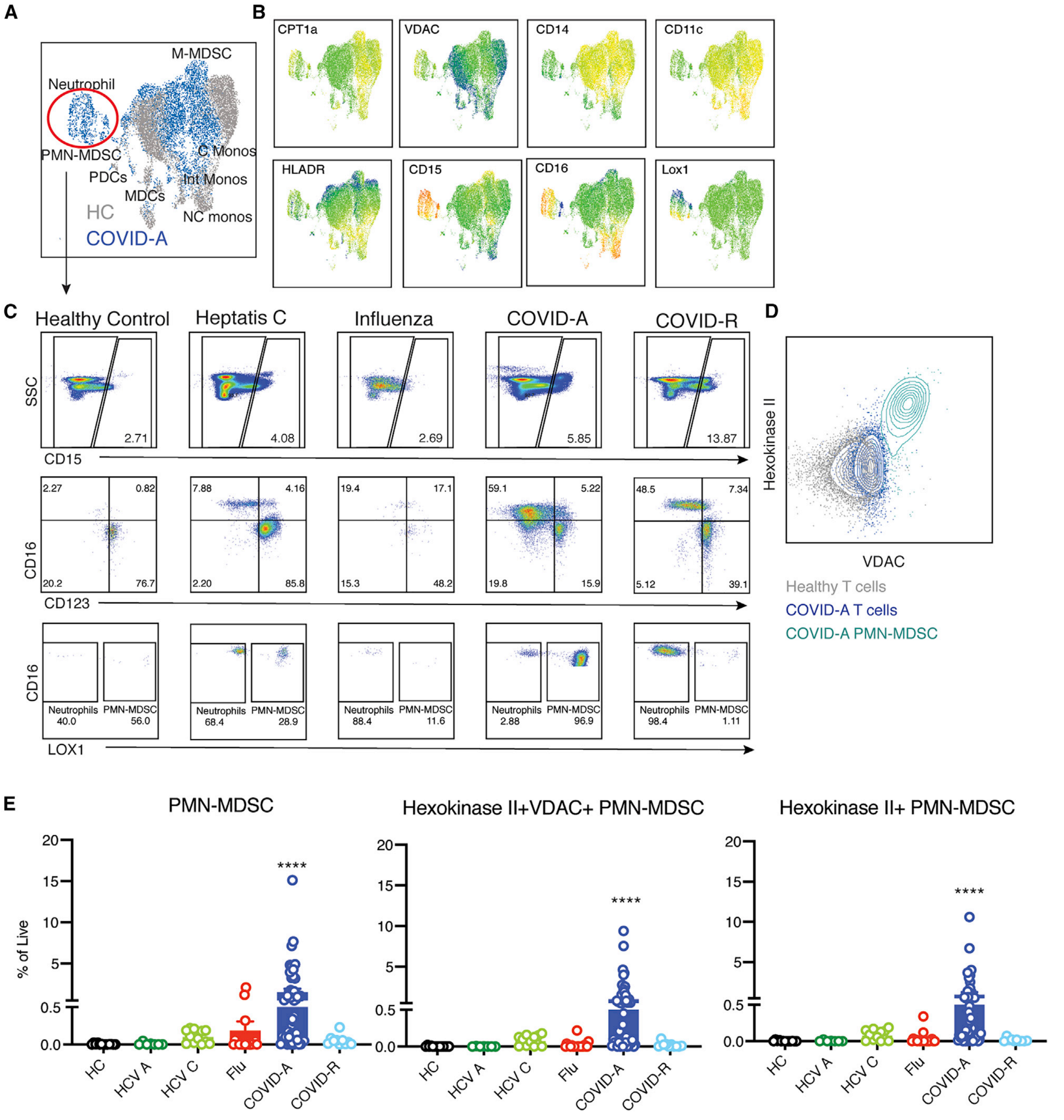

Figure 5. Metabolically distinct granulocytic immunosuppressive myeloid-derived suppressor cells (MDSCs) in PBMCs of COVID-19 patients

(A) Concatenated flow cytometry data depicted as UMAP projection of $CD3^{-}CD19^{-}CD56^{-}$ myeloid cells from HCs (gray) and COVID-A patients (blue).

(B) UMAP projection of MFI heatmap overlays of indicated proteins.

(C) Representative gating of $CD15^{+}$ granulocytic subsets of basophils, eosinophils, neutrophils, and PMN-MDSCs across all disease states studied.

(D) Flow plot comparing HKII and VDAC1 expression in HC T cells (gray), COVID-A T cells (dark blue), and COVID-A PMN-MDSCs (light blue).

(E) Frequency of indicated cell subset as percent of total live cells. Each dot represents one patient sample; significance was tested using unpaired Kruskal-Wallis test compared with HCs. *p < 0.05, **p < 0.01, ***p < 0.001, ****p < 0.0001.

enzyme found within the mitochondrial membrane that is essential for fatty acid oxidation. Additionally, these myeloid cells also expressed high levels of VDAC1 (Figures 6A and 6B). Interestingly, CPT1a has been associated with both inflammasome activation and reactive oxygen species (ROS) production (Hall et al., 2013; Moon et al., 2016), and a potential role for inflammasome

CellPress
OPEN ACCESS

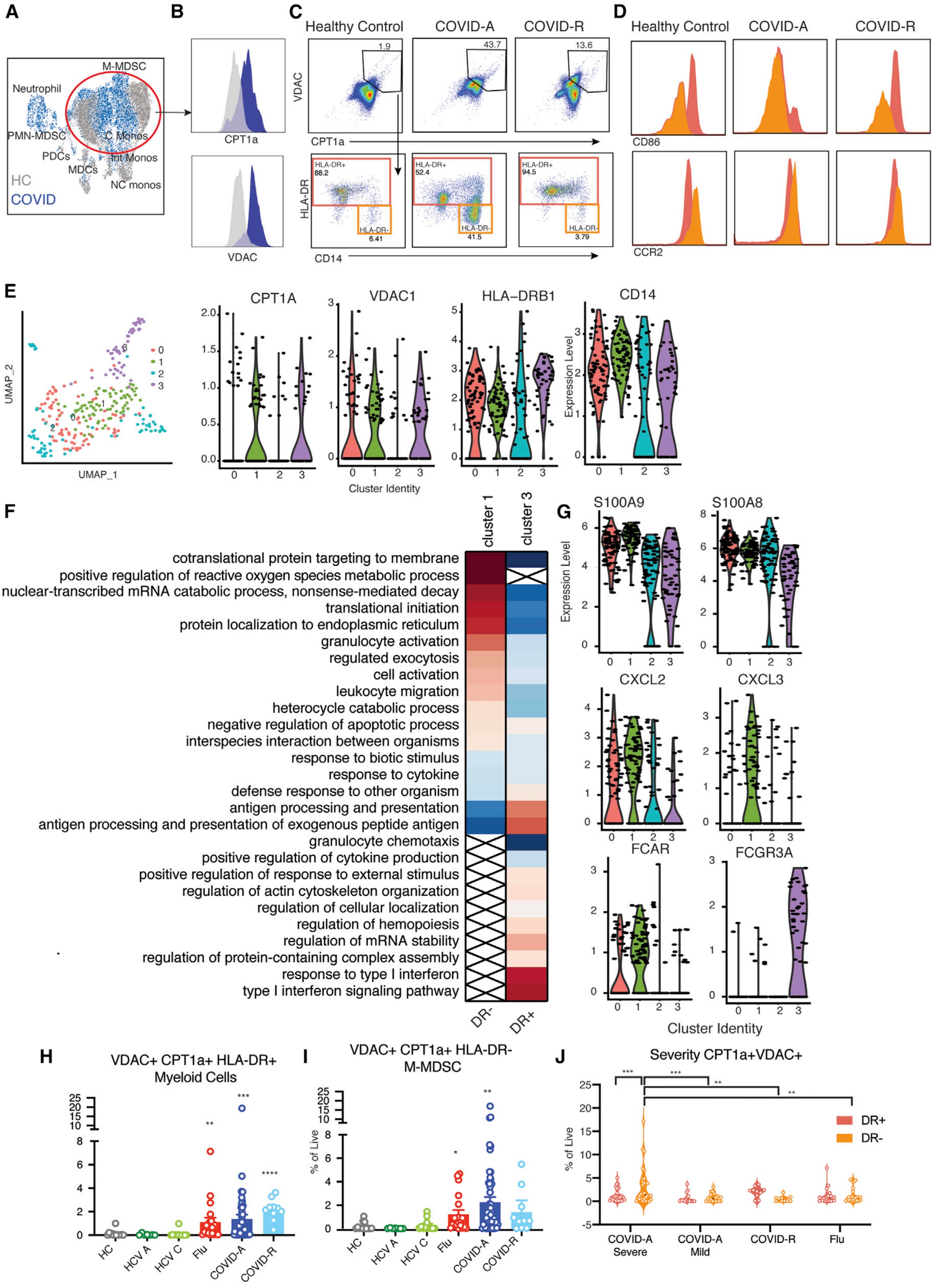

(legend on next page)

activation in contributing to the pathogenesis of SARS-CoV-2 infection has been noted by others (Lucas et al., 2020). When comparing the myeloid cells found in COVID-A versus COVID-R patients, there was a similar metabolic program driven by high expression of CPT1a and VDAC1; however, there was differential expression of HLA-DR (Figure 6C). During acute infection, a large portion of the CPT1a$^+$VDAC1$^+$ myeloid cells were HLA-DR$^-$, and thus classified as M-MDSCs (Bronte et al., 2016) (Figure 6C). A decrease in HLA-DR expression on myeloid cells during COVID-19 infection has been previously noted by other groups as well (Arunachalam et al., 2020; Lucas et al., 2020; Wilk et al., 2020). M-MDSCs are known potent suppressors of T cell responses, and in line with this role of immunosuppression, they expressed diminished levels of costimulatory molecules such as CD86 compared with their HLA-DR$^+$ counterparts (Figure 6D). Further, they expressed elevated levels of CCR2, indicating recent migration out of the bone marrow (Figure 6D).

Conversely, the HLA-DR$^+$ component, composed of both CD14$^+$ and CD16$^+$ monocytes, was elevated during active infection and remained high during recovery (Figure 6C). HLA-DR$^+$ monocytes are capable of producing robust amounts of cytokines and also providing antigen presentation and T cell costimulation, as indicated by the elevated CD86 expression (Figure 6D). The differential expression patterns of these monocytic populations between the infected and recovered patients may represent a shift from immunosuppressive (DR$^-$, MDSCs) to a productive immune response (DR$^+$, stimulatory monocytes).

To better understand the function of the two subsets of CPT1a$^+$VDAC1$^+$ HLA-DR$^-$ cells, we assessed single-cell RNA-seq data from the PBMCs of three COVID-A patients that had high levels of the CPT1a$^+$VDAC1$^+$ myeloid cells, as determined by flow cytometry (Figure 6E). The analysis revealed four distinct clusters (Figure 6E). Clusters 1 and 3 both had elevated levels of VDAC1 and CPT1a, but cluster 1 expressed relatively lower levels of HLA-DR compared with cluster 3 (Figure 6E), corresponding to the two populations of cells identified by flow cytometry. Cluster 3 (HLA-DR$^+$) exhibited gene programs associated with a productive immune response, such as antigen processing/presentation and a type I IFN response (Figure 6F). In contrast, cluster 1, which demonstrated decreased HLA-DR expression and contained the cells that were more abundant in acute infection, expressed gene programs associated with ROS production, exocytosis, and targeting proteins to the membrane surface (Figure 6F). Therefore, the gene programs supported the hypothesis of an increased immunosuppressive myeloid compartment during acute infection, which transitions into an immunostimulatory profile during resolution. Specifically, the HLA-DR$^-$ cluster expressed high levels of genes for secreted alarmins, such as S100A9 and S100A8, and chemokines, such as CXCL2 and CXCL3 (Schulte-Schrepping et al., 2020) (Figure 6G). Overall, our data demonstrate that the immune activating DR$^+$ cells are present in both the COVID-A and COVID-R patients (Figure 6H). However, the DR$^-$ M-MDSC suppressor cells are found in the COVID-A patients, but not the COVID-R patients (Figure 6I).

The dichotomy based on HLA-DR expression of these metabolically unique cells and association with productive or inhibitory immune responses prompted us to examine the relationship between the HLA-DR$^+$ and HLA-DR$^-$ CPT1a$^+$VDAC1$^+$ myeloid cells and disease severity. The CPT1a$^+$VDAC1$^+$ HLA-DR$^-$ cells represent less than 0.5% of total PBMCs of COVID-R patients; however, the percentage of these cells is significantly higher in COVID-A patients requiring mechanical ventilation (on average 3.5% of PBMCs) than in COVID-A patients with less severe disease (0.7% of PBMCs) (Figure 6J). Although we do not observe these cells in the (nonrespiratory) hepatitis C-infected patients, the DR$^-$ cells are present in the influenza patients (Figures 6I and 6J). However, the overall percentage of these cells in the influenza patients is significantly lower than that of the severe COVID-A patients. All influenza patients recovered, and this finding is consistent with the fact that although these patients have been hospitalized, they are more in line with the mild COVID-A patients and, overall, far less ill than the severe COVID-A patients.

The unique T cell and myeloid subsets in the PBMCs of COVID-A patients distinguish disease severity

Finally, although our immune-metabolic approach has defined previously undescribed T cell and myeloid subsets in the COVID-A patients that provide insight into the mechanism of immune dysfunction and pathogenesis, we wondered whether the presence of these cells could be exploited to predict and track severity of disease. To this end, we used a random forest model to identify features most important in distinguishing COVID-19

Figure 6. Identification of metabolically distinct monocytic MDSCs in PBMCs of COVID-19 patients tracked with disease severity

(A) Concatenated flow cytometry data depicted as UMAP projection of CD3$^-$CD19$^-$CD56$^-$ myeloid cells from HCs (gray) and COVID-A patients (blue).

(B) Histogram overlays of MFI for metabolic markers CPT1a and VDAC1 from HCs (gray) or COVID-A patients (blue).

(C) Representative gating of CPT1a$^+$VDAC1$^+$ myeloid cells (gated on CD3$^-$CD19$^-$CD56$^-$ and CD33$^+$) and subset of CPT1a$^+$VDAC1$^+$ cells based on HLA-DR expression.

(D) Histogram overlays of MFI for indicated proteins from CPT1a$^+$VDAC1$^+$HLA-DR$^+$ (red) or CPT1a$^+$VDAC1$^+$HLA-DR$^-$ (orange) cells.

(E) UMAP projection of scRNA-seq of myeloid cells from three COVID patients with detectable CPT1a$^+$VDAC1$^+$ myeloid cells by flow cytometry colored by identified clusters 0–3. Expression of indicated genes within clusters 0–3. Each dot represents a single cell.

(F) Genes identifying cluster 1 (CPT1a$^+$VDAC1$^+$ HLA-DR^{dim}) and cluster 3 (CPT1a$^+$VDAC1$^+$ HLA-DR^{high}) were evaluated for statistical overrepresentation using GO biological processes as gene sets and categorized into higher-level annotation using ReviGO. Heatmap color corresponds to the enrichment score in upregulated genes (red) and downregulated genes (blue); × indicates a non-significant enrichment.

(G) Expression of indicated gene within clusters 0–3. Each dot represents a single cell.

(H and I) Frequency of HLA-DR$^-$ CPT1a$^+$VDAC1$^+$ myeloid cells and HLA-DR$^+$ CPT1a$^+$VDAC1$^+$ myeloid cells as percent of total live cells. Each dot represents one individual; significance was tested using unpaired Kruskal-Wallis test compared with HCs.

(J) Frequency of CPT1a$^+$VDAC1$^+$ DR$^{+/-}$ cells in COVID-A patients was stratified by disease severity (severe = deceased or required mechanical ventilation; mild = hospitalized with low or high flow oxygen). Each dot represents one patient sample; significance was tested using two-way ANOVA comparing either HLA-DR$^+$ versus HLA-DR$^-$ within each category or HLA-DR$^+$ or HLA-DR$^-$ across each category. *$p < 0.05$, **$p < 0.01$, ***$p < 0.001$, ****$p < 0.0001$.

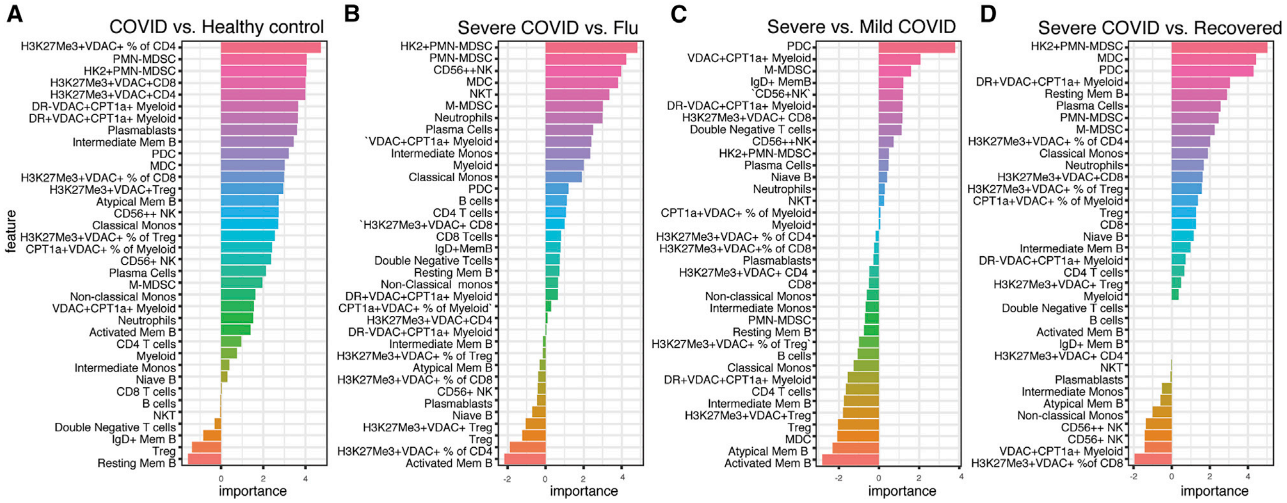

Figure 7. Presence of immune cells with distinct metabolic profiles predicts disease severity

Feature importance of distinguishing COVID-A patients and HCs, COVID-A patients and Flu patients, severe COVID-A patients and COVID-R patients, or severe COVID-A patients and mild COVID-A patients as indicated. Each feature is depicted as a frequency of total live cells, unless otherwise indicated in the case of proportion of cell subset with a specific metabolic phenotype.

patients from healthy controls and influenza patients, or between disease severity and recovery (Figure 7). We found that the proportion of H3K27Me3$^+$VDAC1$^+$ CD4 T cells was highly predictive when comparing healthy controls with COVID-19 patients (Figure 7A). Alternatively, when distinguishing between disease severity or between an otherwise similar respiratory viral disease, the innate compartment was highly informative (Figures 7B and 7C). As previously described, the absence of pDCs was able to distinguish mild from severe COVID-19 (Figure 7C). However, we also found that the HKII$^+$ PMN-MDSC cells we identified were associated with severe disease and could readily discriminate severe COVID from influenza, recovered patients, and healthy controls (Figures 7A, 7B, and 7D). In addition, CPT1a$^+$VDAC1$^+$ myeloid cells (particularly the presence of HLA-DR$^-$ M-MDSCs) were associated with more severe disease (Figure 7C). Finally, because prior publications have shown the association between COVID-19 severity and age, sex, and BMI, we further added these variables to our COVID-19 severity analysis (Figure S7) (Mathew et al., 2020). The feature importance analysis confirmed that in our dataset, sex and BMI are among the most important features in predicting COVID-19 severity. Interestingly, they were less predictive than the percentage of VDAC1$^+$CPT1a$^+$ myeloid cells, pDCs, and H3K27Me3$^+$VDAC1$^+$CD4$^+$ cells (Figure S7B). Adding the percentage of VDAC1$^+$CPT1a$^+$ myeloid cells, pDCs, and H3K27Me3$^+$VDAC1$^+$CD4$^+$ improved prediction of COVID-19 severity compared with basic clinical information (Figure S7C), highlighting the important contribution of the immune cell populations identified to disease severity. This model was validated using an additional 18 COVID-A patients and 5 COVID-R patients not studied in the original cohort (Figure S7D).

DISCUSSION

Considering that metabolic reprogramming plays an integral role in immune cell differentiation and function, we devised an assay to interrogate metabolic programs at a single-cell level. Previous studies examining PBMCs from COVID-19 patients have revealed generalized changes in cellular subsets and cytokines consistent with increased inflammation (Kuri-Cervantes et al., 2020; Laing et al., 2020; Mathew et al., 2020; Wilk et al., 2020). In contrast, by employing our approach, we were will able to identify robust, distinct T cell and myeloid subsets that were unique to the PBMCs of the COVID-A patients. These previously undescribed populations of cells provide important insight into the immune dysfunction and pathogenesis of SARS-CoV-2 infection. Furthermore, as well as defining novel immune cell subsets, our approach reveals a new line of inquiry to understanding immune responses in general during infection, inflammatory diseases, and cancer.

In trying to understand the mechanism of pathogenesis of SARS-CoV-2 infection, there has been much focus on overexuberant immune responses and cytokine storm (Chen et al., 2020; Kuri-Cervantes et al., 2020; Lucas et al., 2020). In contrast, our data have revealed the presence of cells consistent with dysfunctional and suppressive immune responses. T cells play a critical role in both killing virally infected cells and providing help for effective and prolonged anti-viral antibody production (La Gruta and Turner, 2014). Our data reveal the presence of populations of H3K27me3^{hi}VDAC1^{hi} T cells in the COVID-A patients. These cells expressed low levels of Glut1, the glucose transporter necessary to support glycolysis during effector responses. Although increased VDAC1 levels in and of themselves might simply represent increased mitochondrial mass, we observed parameters indicative of mitochondrial dysfunction and apoptosis signaling in these cells, including altered mitochondrial ultrastructure and release of mitochondrial cytochrome *c* into the cytosol. Furthermore, observed loss of cell viability was reversed by treatment with the pan-caspase inhibitor Z-VAD, suggesting caspase-dependent apoptosis. Rescue from cell death was likewise achieved by inhibiting VDAC1 oligomerization, further supporting a mitochondrial mediated cell death program. VDAC1 is the most

prevalent protein in the mitochondrial outer membrane and acts as the main transporter of nucleotides and metabolites across the mitochondrial membrane (Shoshan-Barmatz et al., 2010). By interacting with pro-apoptotic BCL2 family members, VDAC1 oligomerization may allow for pores large enough to release cytochrome *c* into the cytoplasm (Camara et al., 2017). In addition, VDAC1 has also been shown to facilitate BAX/BAK-mediated cell death (Cheng et al., 2003; Shimizu et al., 1999, 2001).

The prevalence of the H3K27me3^{hi}VDAC1^{hi} T cells increased with age, and such cells were observed in all of the patients over 70 years of age in our study. Interestingly, increased age is a known risk factor for severe COVID-19, and aging is associated with a decline in lymphocyte mitochondria fitness and activity (Bratic and Larsson, 2013; Quinn et al., 2019). Additionally, increased prevalence of the H3K27me3^{hi}VDAC1^{hi} T cells correlated with lymphopenia in the COVID-A patients. Lymphopenia has previously been described as a prominent feature of COVID-19, and our data suggest that mechanistically this may be due in part to mitochondrial-induced cell death (Huang et al., 2020). To this end, our data support direct T cell death, as opposed to trafficking to inflamed organs such as the lung (Vabret et al., 2020). Further, this dysregulated T cell immunity could contribute to a lack of or waning protective immunity or impair the functionality of pre-existing cross-reactive T cell immunity (Grifoni et al., 2020; Sekine et al., 2020). Alternatively, it is possible that increased cell death of T cells is driving some of the dysregulated inflammation and autoimmune-like features characteristic of COVID-19. Finally, our data suggest that therapeutically targeting mitochondrial (metabolic) dysfunction might represent a successful strategy for abrogating disease.

The precise inflammatory mediators leading to the generation of the H3K27me3^{hi}VDAC1^{hi} T cells has yet to be determined. Several lines of evidence indicate that this T cell phenotype is driven by more than the cytokine environment or TCR stimulation alone. Memory T cell subsets are more responsive to secondary cytokine signaling compared with naive T cells (Lauvau et al., 2016); however, elevated VDAC1 and H3K27me3 expression were found equally in the naive and memory compartment. Further, there was no expansion of specific TCR clones within the H3K27me3^{hi}VDAC1^{hi} phenotype, indicating this is likely not driven by recent antigen exposure. Because most of the hospitalized COVID-A patients were hypoxic, requiring ntal oxygen, an interesting possibility is that SARS-CoV-2-induced inflammation in the setting of hypoxia contributes to the generation of these dysfunctional T cells. Indeed, oxidative phosphorylation is required for the maintenance of T cell function and is the predominant metabolic pathway utilized by resting T cells (Pearce et al., 2013). Thus, COVID-19 hypoxia combined with a specific inflammatory environment may lead to this metabolically dysregulated T cell phenotype.

Previous studies have demonstrated increased inflammation and activation of myeloid cells in COVID-19 patients (Arunachalam et al., 2020; Kuri-Cervantes et al., 2020; Lucas et al., 2020). However, it still remains unclear which of these responses are protective and which are pathogenic. Our identification of unique myeloid populations in the COVID-A patients shed light on the role of these cells. HKII^{+} VDAC1^{+} PMN-MDSC were exclusively upregulated in the COVID-A patients, suggesting that this population of cells is either contributing to pathogenesis or the consequence of dysregulated inflammatory responses. On the other hand, CPT1a^{+}VDAC1^{+}DR^{+} monocytic cells were found in both the acute and recovered patients. DR^{+} monocytes have previously been shown to be associated with productive immune responses in other respiratory viral infections (Vangeti et al., 2018), and single-cell RNA sequence analysis revealed increased antigen processing/presentation and type I IFN responses in such cells. In contrast, the CPT1a^{+}VDAC1^{+}DR^{-} M-MDSCs were found exclusively in the COVID-A patients and further, the frequency of these cells was positively correlated with severity of disease. As such, the presence of these suppressor cells seems to be indicative of dysregulated inflammation. Finally, CPT1a has been associated with inflammasome activation, which has been observed in COVID-19 patients, and its role in fatty acid oxidation supports modulators of either fatty acid oxidation or inflammasome signaling as potentially novel therapeutic targets to mitigate disease.

Finally, the unique cellular subsets described herein represent potentially potent biomarkers to predict and track severity of disease. The importance of the described cell populations is highlighted by their ability to robustly contribute to distinguishing severe and mild COVID-19 and COVID-A from other viral infections. Consequently, tracking these unique cells might identify those at highest risk for disease progression and provide important criteria for enrollment into clinical trials, as well as provide a surrogate marker for tracking efficacy of new potential treatments. Together, our data demonstrate the utility of broad immunometabolic phenotyping to identify subsets of immune cells that have the potential to not only provide insight into disease pathogenesis and predict severity of disease but also, importantly, to define novel metabolic targets for treatment.

Limitations of study

Institutional safety protocols led to certain experimental limitations of our study. For example, mitochondrial studies had to be performed on fixed cells limiting the use of certain dyes and limiting our ability to extend the depth of our studies concerning mitochondrial dysfunction. Nonetheless, we employed three different microscopic techniques (Figure 3) to prove this point. Along these lines, the fact that VBIT-4, the inhibitor of VDAC1 aggregation, promotes survival in the T cells from the COVID-19 patients, but not healthy controls, supports a role for VDAC1 in promoting cell death. Although we also observe cytochrome *c* in the cytoplasm of such T cells, we have not definitively proven that VDAC1 pore formation leading to cytochrome *c* in the cytoplasm is the definitive/sole mechanism of cell death. Additionally, while our study is highlighted by insightful comparisons between SARS-CoV-2-infected patients and hospitalized influenza-infected patients, limited availability of cells from the influenza cohort precluded more in-depth functional comparisons between the two sets of patients.

STAR★METHODS

Detailed methods are provided in the online version of this paper and include the following:

- KEY RESOURCES TABLE

- RESOURCE AVAILABILITY
  - Lead contact
  - Materials availability
  - Data and code availability
- EXPERIMENTAL MODEL AND SUBJECT DETAILS
  - Study Participants
  - Sample processing and PBMC isolation
- METHOD DETAILS
  - Immuno-metabolic *ex vivo* flow cytometry staining
  - FACS cell sorting & TCRseq
  - Single-cell RNaseq
  - Electron Microscopy
  - Immunofluorescence
  - *In vitro* T cell stimulations
  - Feature importance and prediction analysis
- QUANTIFICATION AND STATISTICAL ANALYSIS

SUPPLEMENTAL INFORMATION

Supplemental information can be found online at https://doi.org/10.1016/j.celrep.2021.108863.

ACKNOWLEDGMENTS

We would like to acknowledge the contribution of the Johns Hopkins IVAR team and the JH-EPICS team and assistance for clinical data coordination and retrieval from the Core for Clinical Research Data Acquisition. The specimens from COVID-19 patients utilized for this study were part of the Johns Hopkins Biospecimen Repository, which is based on the contribution of many patients, their families, research teams, and clinicians. We would also like to acknowledge the Electron Microscopy Lab, Department of Pathology, and the Experimental and Computational Genomics Core at the Sidney Kimmel Comprehensive Cancer Center, Johns Hopkins University (JHU) for help in this study. Likewise, we would like to thank the patients and teams of the Baltimore Before and After Acute Study of Hepatitis (BBAASH), the Johns Hopkins Center of Excellence in Influenza Research and Surveillance (JH CEIRS), Biomedical Advanced Research and Development Authority (BARDA), and the Convalescent Plasma Donor Study. Funding for this work was provided by a Johns Hopkins University Provost Research Grant, The Bill and Melinda Gates Foundation (134582), National Institutes of Health (NIH) Centers of Excellence in Influenza Research and Surveillance (HHSN272201400007C), and NIH (P41EB028239 to J.D.P.). The study was supported in part by a cooperative agreement between JHU and the Division of Intramural Research, National Institute of Allergy and Infectious Diseases (NIAID), NIH, as well as extramural support from the US Department of Health and Human Services Biomedical Advanced Research and Development Authority (BARDA; grant number IDSEP160031-01-00) and NIAID (R01AI120938, R01AI120938S1, R01AI128779, R01-AI153349, R01-AI145435-A1, R21-AI149760, and U19A1088791). Contract HHSN272201400007C was awarded to A.A.R.T., the Johns Hopkins Center for Influenza Research and Surveillance (JHCEIRS) at Johns Hopkins University. Confocal microscopy images acquired from Zeis LSM880 were supported by National Institute of General Medical Sciences (NIGMS) of the NIH (S10OD023548 to JHUSOM Microscope Facility). The Core for Clinical Research Data Acquisition is supported in part by the Johns Hopkins Institute for Clinical and Translational Research (UL1TR001079). The computational work by W.Z. and H.J. was supported in part by the National Human Genome Research Institute of the NIH grant R01HG009518.

AUTHOR CONTRIBUTIONS

Conceptualization, data curation, formal analysis, investigation, methodology, validation, visualization, writing – original draft, review & editing, E.A.T. and K.C.; investigation, A.A.O.; formal analysis and visualization, W.Z., A.V., A.H.-B., N.R.B., I.-H.S., R.W., A.Z.R., and H.J.; sample acquisition, R.R., K.F., L.S., K.S.-S., E.M.B., A.D.R., and A.A.R.T.; funding acquisition, M.H., K.S., A.P., F.R.D., and S.Y.; conceptualization, funding acquisition, methodology, project administration, resources, writing – review & editing, A.L.C. and J.D.P.

DECLARATION OF INTERESTS

J.D.P. is a scientific founder, a paid consultant, and has equity in Dracen Pharmaceuticals.

Received: October 5, 2020
Revised: December 17, 2020
Accepted: February 22, 2021
Published: February 26, 2021

REFERENCES

Abu-Hamad, S., Arbel, N., Calo, D., Arzoine, L., Israelson, A., Keinan, N., Ben-Romano, R., Friedman, O., and Shoshan-Barmatz, V. (2009). The VDAC1 N-terminus is essential both for apoptosis and the protective effect of anti-apoptotic proteins. J. Cell Sci. *122*, 1906–1916.

Arunachalam, P.S., Wimmers, F., Mok, C.K.P., Perera, R.A.P.M., Scott, M., Hagan, T., Sigal, N., Feng, Y., Bristow, L., Tak-Yin Tsang, O., et al. (2020). Systems biological assessment of immunity to mild versus severe COVID-19 infection in humans. Science *369*, 1210–1220.

Ben-Hail, D., Begas-Shvartz, R., Shalev, M., Shteinfer-Kuzmine, A., Gruzman, A., Reina, S., De Pinto, V., and Shoshan-Barmatz, V. (2016). Novel Compounds Targeting the Mitochondrial Protein VDAC1 Inhibit Apoptosis and Protect against Mitochondrial Dysfunction. J. Biol. Chem. *291*, 24986–25003.

Bengsch, B., Johnson, A.L., Kurachi, M., Odorizzi, P.M., Pauken, K.E., Attanasio, J., Stelekati, E., McLane, L.M., Paley, M.A., Delgoffe, G.M., and Wherry, E.J. (2016). Bioenergetic Insufficiencies Due to Metabolic Alterations Regulated by the Inhibitory Receptor PD-1 Are an Early Driver of CD8(+) T Cell Exhaustion. Immunity *45*, 358–373.

Bratic, A., and Larsson, N.G. (2013). The role of mitochondria in aging. J. Clin. Invest. *123*, 951–957.

Bronte, V., Brandau, S., Chen, S.H., Colombo, M.P., Frey, A.B., Greten, T.F., Mandruzzato, S., Murray, P.J., Ochoa, A., Ostrand-Rosenberg, S., et al. (2016). Recommendations for myeloid-derived suppressor cell nomenclature and characterization standards. Nat. Commun. *7*, 12150.

Buck, M.D., O'Sullivan, D., and Pearce, E.L. (2015). T cell metabolism drives immunity. J. Exp. Med. *212*, 1345–1360.

Buck, M.D., Sowell, R.T., Kaech, S.M., and Pearce, E.L. (2017). Metabolic Instruction of Immunity. Cell *169*, 570–586.

Camara, A.K.S., Zhou, Y., Wen, P.C., Tajkhorshid, E., and Kwok, W.M. (2017). Mitochondrial VDAC1: A Key Gatekeeper as Potential Therapeutic Target. Front. Physiol. *8*, 460.

Carlson, C.S., Emerson, R.O., Sherwood, A.M., Desmarais, C., Chung, M.W., Parsons, J.M., Steen, M.S., LaMadrid-Herrmannsfeldt, M.A., Williamson, D.W., Livingston, R.J., et al. (2013). Using synthetic templates to design an unbiased multiplex PCR assay. Nat. Commun. *4*, 2680.

Chen, G., Wu, D., Guo, W., Cao, Y., Huang, D., Wang, H., Wang, T., Zhang, X., Chen, H., Yu, H., et al. (2020). Clinical and immunological features of severe and moderate coronavirus disease 2019. J. Clin. Invest. *130*, 2620–2629.

Cheng, E.H., Sheiko, T.V., Fisher, J.K., Craigen, W.J., and Korsmeyer, S.J. (2003). VDAC2 inhibits BAK activation and mitochondrial apoptosis. Science *301*, 513–517.

Cox, A.L., Netski, D.M., Mosbruger, T., Sherman, S.G., Strathdee, S., Ompad, D., Vlahov, D., Chien, D., Shyamala, V., Ray, S.C., and Thomas, D.L. (2005). Prospective evaluation of community-acquired acute-phase hepatitis C virus infection. Clin. Infect. Dis. *40*, 951–958.

Dong, E., Du, H., and Gardner, L. (2020). An interactive web-based dashboard to track COVID-19 in real time. Lancet Infect. Dis. *20*, 533–534.

Dugas, A.F., Hsieh, Y.H., LoVecchio, F., Moran, G.J., Steele, M.T., Talan, D.A., and Rothman, R.E.; Emergency Department National Influenza Network

Investigators (2020). Derivation and Validation of a Clinical Decision Guideline for Influenza Testing in 4 US Emergency Departments. Clin. Infect. Dis. *70*, 49–58.

Garrido, C., Galluzzi, L., Brunet, M., Puig, P.E., Didelot, C., and Kroemer, G. (2006). Mechanisms of cytochrome c release from mitochondria. Cell Death Differ. *13*, 1423–1433.

Grifoni, A., Weiskopf, D., Ramirez, S.I., Mateus, J., Dan, J.M., Moderbacher, C.R., Rawlings, S.A., Sutherland, A., Premkumar, L., Jadi, R.S., et al. (2020). Targets of T Cell Responses to SARS-CoV-2 Coronavirus in Humans with COVID-19 Disease and Unexposed Individuals. Cell *181*, 1489–1501.e15.

Hall, C.J., Boyle, R.H., Astin, J.W., Flores, M.V., Oehlers, S.H., Sanderson, L.E., Ellett, F., Lieschke, G.J., Crosier, K.E., and Crosier, P.S. (2013). Immunoresponsive gene 1 augments bactericidal activity of macrophage-lineage cells by regulating β-oxidation-dependent mitochondrial ROS production. Cell Metab. *18*, 265–278.

Henson, S.M., and Akbar, A.N. (2009). KLRG1—more than a marker for T cell senescence. Age (Dordr.) *31*, 285–291.

Huang, C., Wang, Y., Li, X., Ren, L., Zhao, J., Hu, Y., Zhang, L., Fan, G., Xu, J., Gu, X., et al. (2020). Clinical features of patients infected with 2019 novel coronavirus in Wuhan, China. Lancet *395*, 497–506.

Kim, J., Gupta, R., Blanco, L.P., Yang, S., Shteinfer-Kuzmine, A., Wang, K., Zhu, J., Yoon, H.E., Wang, X., Kerkhofs, M., et al. (2019). VDAC oligomers form mitochondrial pores to release mtDNA fragments and promote lupus-like disease. Science *366*, 1531–1536.

Klein, S.L., Pekosz, A., Park, H.-S., Ursin, R.L., Shapiro, J.R., Benner, S.E., Littlefield, K., Kumar, S., Naik, H.M., Betenbaugh, M.J., et al. (2020). Sex, age, and hospitalization drive antibody responses in a COVID-19 convalescent plasma donor population. J. Clin. Invest. *130*, 6141–6150.

Kuhn, M. (2020). caret: Classification and Regression Training. R package version 60-85.. https://cran.r-project.org/web/packages/caret/index.html.

Kuri-Cervantes, L., Pampena, M.B., Meng, W., Rosenfeld, A.M., Ittner, C.A.G., Weisman, A.R., Agyekum, R.S., Mathew, D., Baxter, A.E., Vella, L.A., et al. (2020). Comprehensive mapping of immune perturbations associated with severe COVID-19. Sci. Immunol. *5*, eabd7114.

La Gruta, N.L., and Turner, S.J. (2014). T cell mediated immunity to influenza: mechanisms of viral control. Trends Immunol. *35*, 396–402.

Laing, A.G., Lorenc, A., del Molino del Barrio, I., Das, A., Fish, M., Monin, L., Muñoz-Ruiz, M., McKenzie, D.R., Hayday, T.S., Francos-Quijorna, I., et al. (2020). A dynamic COVID-19 immune signature includes associations with poor prognosis. Nat. Med. *26*, 1623–1635.

Lauvau, G., Boutet, M., Williams, T.M., Chin, S.S., and Chorro, L. (2016). Memory CD8(+) T Cells: Innate-Like Sensors and Orchestrators of Protection. Trends Immunol. *37*, 375–385.

Lucas, C., Wong, P., Klein, J., Castro, T.B.R., Silva, J., Sundaram, M., Ellingson, M.K., Mao, T., Oh, J.E., Israelow, B., et al. (2020). Longitudinal analyses reveal immunological misfiring in severe COVID-19. Nature *585*, 463–469.

Mathew, D., Giles, J.R., Baxter, A.E., Oldridge, D.A., Greenplate, A.R., Wu, J.E., Alanio, C., Kuri-Cervantes, L., Pampena, M.B., D'Andrea, K., et al.; UPenn COVID Processing Unit (2020). Deep immune profiling of COVID-19 patients reveals distinct immunotypes with therapeutic implications. Science *369*, eabc8511.

Mazzoni, A., Salvati, L., Maggi, L., Capone, M., Vanni, A., Spinicci, M., Mencarini, J., Caporale, R., Peruzzi, B., Antonelli, A., et al. (2020). Impaired immune cell cytotoxicity in severe COVID-19 is IL-6 dependent. J. Clin. Invest. *130*, 4694–4703.

Moon, J.S., Nakahira, K., Chung, K.P., DeNicola, G.M., Koo, M.J., Pabón, M.A., Rooney, K.T., Yoon, J.H., Ryter, S.W., Stout-Delgado, H., and Choi, A.M. (2016). NOX4-dependent fatty acid oxidation promotes NLRP3 inflammasome activation in macrophages. Nat. Med. *22*, 1002–1012.

Patel, C.H., Leone, R.D., Horton, M.R., and Powell, J.D. (2019). Targeting metabolism to regulate immune responses in autoimmunity and cancer. Nat. Rev. Drug Discov. *18*, 669–688.

Pearce, E.L., and Shen, H. (2006). Making sense of inflammation, epigenetics, and memory CD8+ T-cell differentiation in the context of infection. Immunol. Rev. *211*, 197–202.

Pearce, E.L., Poffenberger, M.C., Chang, C.H., and Jones, R.G. (2013). Fueling immunity: insights into metabolism and lymphocyte function. Science *342*, 1242454.

Quinn, K.M., Palchaudhuri, R., Palmer, C.S., and La Gruta, N.L. (2019). The clock is ticking: the impact of ageing on T cell metabolism. Clin. Transl. Immunology *8*, e01091.

Schulte-Schrepping, J., Reusch, N., Paclik, D., Baßler, K., Schlickeiser, S., Zhang, B., Krämer, B., Krammer, T., Brumhard, S., Bonaguro, L., et al. (2020). Severe COVID-19 Is Marked by a Dysregulated Myeloid Cell Compartment. Cell *182*, 1419–1440.e23.

Sekine, T., Perez-Potti, A., Rivera-Ballesteros, O., Strålin, K., Gorin, J.-B., Olsson, A., Llewellyn-Lacey, S., Kamal, H., Bogdanovic, G., Muschiol, S., et al. (2020). Robust T cell immunity in convalescent individuals with asymptomatic or mild COVID-19. Cell *183*, 158–168.e14.

Shimizu, S., Narita, M., and Tsujimoto, Y. (1999). Bcl-2 family proteins regulate the release of apoptogenic cytochrome c by the mitochondrial channel VDAC. Nature *399*, 483–487.

Shimizu, S., Ide, T., Yanagida, T., and Tsujimoto, Y. (2000). Electrophysiological study of a novel large pore formed by Bax and the voltage-dependent anion channel that is permeable to cytochrome c. J. Biol. Chem. *275*, 12321–12325.

Shimizu, S., Matsuoka, Y., Shinohara, Y., Yoneda, Y., and Tsujimoto, Y. (2001). Essential role of voltage-dependent anion channel in various forms of apoptosis in mammalian cells. J. Cell Biol. *152*, 237–250.

Shoshan-Barmatz, V., Zakar, M., Rosenthal, K., and Abu-Hamad, S. (2009). Key regions of VDAC1 functioning in apoptosis induction and regulation by hexokinase. Biochim. Biophys. Acta *1787*, 421–430.

Shoshan-Barmatz, V., De Pinto, V., Zweckstetter, M., Raviv, Z., Keinan, N., and Arbel, N. (2010). VDAC, a multi-functional mitochondrial protein regulating cell life and death. Mol. Aspects Med. *31*, 227–285.

Silvestre-Roig, C., Fridlender, Z.G., Glogauer, M., and Scapini, P. (2019). Neutrophil Diversity in Health and Disease. Trends Immunol. *40*, 565–583.

Vabret, N., Britton, G.J., Gruber, C., Hegde, S., Kim, J., Kuksin, M., Levantovsky, R., Malle, L., Moreira, A., Park, M.D., et al.; Sinai Immunology Review Project (2020). Immunology of COVID-19: Current State of the Science. Immunity *52*, 910–941.

Vangeti, S., Yu, M., and Smed-Sörensen, A. (2018). Respiratory Mononuclear Phagocytes in Human Influenza A Virus Infection: Their Role in Immune Protection and As Targets of the Virus. Front. Immunol. *9*, 1521.

Wang, T.S., Coppens, I., Saorin, A., Brady, N.R., and Hamacher-Brady, A. (2020). Endolysosomal Targeting of Mitochondria Is Integral to BAX-Mediated Mitochondrial Permeabilization during Apoptosis Signaling. Dev. Cell *53*, 627–645.e7.

Wilk, A.J., Rustagi, A., Zhao, N.Q., Roque, J., Martínez-Colón, G.J., McKechnie, J.L., Ivison, G.T., Ranganath, T., Vergara, R., Hollis, T., et al. (2020). A single-cell atlas of the peripheral immune response in patients with severe COVID-19. Nat. Med. *26*, 1070–1076.

Zhang, J.Y., Wang, X.M., Xing, X., Xu, Z., Zhang, C., Song, J.W., Fan, X., Xia, P., Fu, J.L., Wang, S.Y., et al. (2020). Single-cell landscape of immunological responses in patients with COVID-19. Nat. Immunol. *21*, 1107–1118.

Zhou, J., Nefedova, Y., Lei, A., and Gabrilovich, D. (2018). Neutrophils and PMN-MDSC: Their biological role and interaction with stromal cells. Semin. Immunol. *35*, 19–28.

CellPress
OPEN ACCESS

STAR★METHODS

KEY RESOURCES TABLE

REAGENT or RESOURCE	SOURCE	IDENTIFIER
Antibodies		
Anti-human CD3 BV786	BD Biosciences	Cat# 563800
Anti-human CD8 BV480	BD Biosciences	Cat# 566121
Anti-human CD45RA BV570	Biolegend	Cat# 304132
Anti-human CCR7 BV650	Biolegend	Cat# 353234
Anti-human CD25 BV510	BD Biosciences	Cat# 563352
Anti-human HLA-DR BV750	Biolegend	Cat# 307672
Anti-human CXCR3 BV605	Biolegend	Cat# 353728
Anti-human PD-1 BV711	Biolegend	Cat# 329928
Anti-human CD4 PE-Cy5	Biolegend	Cat# 317412
Anti-human CD69 PE-Cy5.5	ThermoFisher Scientific	Cat# MHCD6918
Anti-human KLRG1 PE-CF594	BD Biosciences	Cat# 565393
Anti-human CD49a APC	Biolegend	Cat# 328314
Anti-human CD19 APC-Cy7	Biolegend	Cat# 363010
Anti-human CD56 APC-Cy7	Biolegend	Cat# 362512
Anti-human FoxP3 PacBlue	Biolegend	Cat# 320116
Anti-human Tomm20 AF405	Abcam	Cat# ab210047
Anti-human VDAC1	Abcam	Cat# ab14734
Anti-human CPT1a AF488	Abcam	Cat# ab171449
Anti-human Ki67 PE-Cy7	Biolegend	Cat# 350526
Anti-human H3K27me3 PE	CST	Cat# 40724
Anti-human HK2 AF680	Abcam	Cat# ab228819
Anti-human GLUT1 AF647	Abcam	Cat# ab195020
Anti-human CD14 BV605	Biolegend	Cat# 301834
Anti-human CD16 BV785	Biolegend	Cat# 302046
Anti-human CD33 BV570	Biolegend	Cat# 303417
Anti-human CD11c BV480	BD Biosciences	Cat# 74392
Anti-human CCR2 BV510	Biolegend	Cat# 357218
Anti-human CD40 PacBlue	Biolegend	Cat# 334320
Anti-human CD38 BV711	BD Biosciences	Cat# 563965
Anti-human CD86 BV650	Biolegend	Cat# 305428
Anti-human IgD BB790	BD Biosciences	Cat# Custom order
Anti-human CD27 PE-CF594	BD Biosciences	Cat# 562297
Anti-human CD21 PE-Cy5	BD Biosciences	Cat# 551064
Anti-human CD138	Biolegend	Cat# 356502
Anti-human CD15 PE-Cy7	Biolegend	Cat# 301924
Anti-human LOX1 PE	Biolegend	Cat# 358604
Anti-human CD123 APC	Biolegend	Cat# 306012
Anti-human CD3 APC-Cy7	Biolegend	Cat# 344818
Anti-human Cytochrome *c*	BD Biosciences	Cat# 556432
Anti-human CD3	Dako	Cat# A0452
Anti-human CD3 FITC	BD Biosciences	Cat# 349201
goat anti-mouse AF488 IgG	ThermoFisher Scientific	Cat# A-11017

(*Continued on next page*)

Continued

REAGENT or RESOURCE	SOURCE	IDENTIFIER
Chemicals, peptides, and recombinant proteins		
Z-VAD-FMK	Cell Signaling Technology	Cat# 60332S
VBIT-4	Fischer Scientific	Cat# NC1761210
RPMI 1640 + L-Glutamine	GIBCO	Cat# 11875-085
Lymphocyte Separation Medium	Corning	Cat# 25-072-CI
Fetal Bovine Serum	Atlanta Biologicals	Cat# S11150
4% paraformaldehyde methanol free	ThermoFisher Scientific	Cat# 28906
Poly-D Lysine	Sigma-Aldrich	Cat# P0899
Critical commercial assays		
Dylight 680 Conjugation Kit (Fast Lightening Link)	Abcam	Cat# ab201804
Alexa Fluor 532 Antibody Labeling Kit	ThermoFisher Scientific	Cat# A20182
PE/Cy5.5® Conjugation Kit - Lightning-Link®	Abcam	Cat# ab102899
Zombie NIR Fixable Viability kit	Biolegend	Cat# 423106
Live/Dead Fixable Aqua	ThermoFisher Scientific	Cat# L34966
Brilliant Stain Buffer Plus	BD Biosciences	Cat# 566385
eBioscience Foxp3 / Transcription Factor Staining Buffer Set	ThermoFisher Scientific	Cat# 00-5523-00
Human BD Fc Block	BD Biosciences	Cat# 564220
Mouse Ig Kappa Comp Beads	BD Biosciences	Cat #552843
MitoTracker Deep Red Dye	ThermoFisher Scientific	Cat# M22426
Slow Fade Diamond anti-fade reagent with DAPI	Invitrogen	Cat# S36964
QIAamp DNA Micro Kit	QIAGEN	Cat# 56304
Chromium Next GEM Single Cell 5′ Library and Gel Bead Kit v1.1, 16 rxns	10x Genomics	1000165
Chromium Next GEM Chip G Single Cell Kit, 48 rxns	10x Genomics	1000120
Chromium Single Cell V(D)J Enrichment Kit, Human T Cell, 96 rxns	10x Genomics	1000005
Chromium Single Cell V(D)J Enrichment Kit, Human B Cell, 96 rxns	10x Genomics	1000016
Single Index Kit T Set A, 96 rxn	10x Genomics	1000213
Deposited data		
Raw and processed RNA-seq data	NCBI GEO	GSE166992
Software and algorithms		
FlowJo software (Tree Star, Inc.)	FlowJo LLC	https://www.flowjo.com/
GraphPad Prism V9	GraphPad Software	https://www.graphpad.com/
Fiji		https://fiji.sc/Fiji
Zen Black software	Zeiss	https://www.zeiss.com/corporate/int/home.html
PANTHER (v. 15.0)	Gene Ontology Phylogenetic Annotations Project	Version 15.0; http://pantherdb.org/
JMP 14 Pro	SAS	https://www.jmp.com/en_us/home.html
Cellranger software (v.3.1.0)	10x genomics	Version 3.1.0
Seurat package (v.3.1)	Satija Lab	Version 3.1; https://satijalab.org/seurat/index.html

RESOURCE AVAILABILITY

Lead contact

Further information and requests for resources and reagents should be directed to and will be fulfilled by the Lead Contact, Jonathan Powell (jpowell@jhmi.edu).

Materials availability

This study did not generate new unique reagents.

Data and code availability

The accession number for the sequencing data reported in this paper is GEO: GSE166992.

EXPERIMENTAL MODEL AND SUBJECT DETAILS

Study Participants

Patients diagnosed with COVID-19 by positive SARS-CoV-2 RNA testing through the Johns Hopkins Healthcare System were enrolled in a protocol designed to generate a biospecimen repository linked to clinical data for investigation (Johns Hopkins Medicine (JHM) IRB 00245545) and another for analysis of research questions specific to immunology (JHM IRB 00255162). Subjects identified as SARS-CoV-2 PCR positive consented to study participation and for clinical information to be linked to their study subject identification number. Subjects are categorized by maximum COVID-19 disease severity score using four groups; minimal oxygen required, high flow nasal cannula required, intubation with survival, and death. Samples, including blood for processing into serum, plasma and PBMC, urine, and swabs of the nasopharyngeal, oropharyngeal, and crevicular spaces were obtained as close to admission as feasible (day 0), 3 and 7 days later, weekly after day 7, and after discharge at regular intervals beginning at day 28 following study entry. Demographic information, clinical laboratory test results, ICD-10 coded diagnoses recorded in the patients' records (comorbidities), medication lists, body mass index, and other clinical parameters were linked to data for all subjects in the study. Subjects in the study are 50% male and 50% female with a mean age of 59.7 years (range 20-82). Detailed demographic information can be found in Table S2. For this study, COVID-19 infected participants on immunosuppressive medications other than inhaled steroids at the time of enrollment were excluded. Following JHM IRB approval, PBMC samples were obtained under informed consent from: HCV infected patients (JHM IRB NA_0004638), hospitalized patients infected with influenza in 2019 (JHM IRB 00091667) and SARS-CoV-2 convalescent plasma donors (JHM IRB 00248402, JHM IRB 00250798) as previously described (Cox et al., 2005; Dugas et al., 2020; Klein et al., 2020) for comparison to hospitalized acutely infected COVID-19 patients in this study. Acute HCV infected patients are 33% male and 67% female with a mean age of 25.8 (range 24-28), chronic HCV patients are 70% male and 30% female with a mean age of 30.5 (range 26-35), hospitalized influenza patients are 43% male and 57% female with a mean age of 46.4 (range 22-89), and convalescent plasma donors (recovered COVID-19) are 60% male and 40% female with a mean age of 47.8 (range 18-81). Demographic information for these patients can be found in Table S3.

Sample processing and PBMC isolation

Peripheral blood was collected from hospitalized COVID-19 patients upon enrollment in the study at day 0 for isolation of serum, plasma and peripheral blood mononuclear cells (PBMCs). When possible, patients who remained in the hospital were also sampled consecutively at day 3 and day 7 post-enrollment. Blood processing was performed in BSL2+ laboratory conditions as approved following safety assessments. Blood was centrifuged at 400 x *g* for 5 min to separate cells from plasma. Cells were resuspended in RPMI, underlaid with Ficoll and centrifuged at 400 x *g* for 30 min without break at room temperature. The PBMC layer was then washed twice in RPMI and PBMCs were viably cryopreserved in FBS + 10% DMSO for future use.

METHOD DETAILS

Immuno-metabolic *ex vivo* flow cytometry staining

All flow cytometry antibodies used for phenotypic and metabolic analysis can be found in table S1. PBMCs from hospitalized COVID-19 patients, hospitalized flu patients, COVID-19 convalescent plasma donors (recovered) and healthy controls were used for phenotypic and metabolic assessment. Cryopreserved PBMCs were thawed in RPMI (GIBCO) + 50% FBS (Atlanta Biologicals). Cells were washed once in PBS and immediately stained for viability with Biolegend Live/Dead Zombie NIR Fixable Viability Dye and BD Fc Block™ for 10 min at room temperature. Cell surface staining was performed in 100uL of 20% BD Horizon™ Brilliant Stain Buffer + PBS with surface stain antibody cocktail for 20 min at room temperature. Cells were fixed and permeabilized with eBioscience™ FoxP3/Transcription Factor Staining kit 1x Fixation/Permeabilization reagent for 20 min at room temperature. Cells were washed with 1x Permeabilization/Wash buffer. Intracellular staining (ICS) was performed in 100uL 1x Permeabilization/Wash buffer with ICS antibody cocktail for 45 min at room temperature. Cells were washed once with Permeabilization/Wash buffer then resuspended in 1% Paraformaldehyde for acquisition by flow. Samples were run on a 3

laser Cytek Aurora spectral flow cytometer. FCS files were analyzed using Flowjo v10 (10.6.2.) software. Manual gating strategies for both the T cell and B cell/Myeloid panels can be found in Figure S1. High-dimensional unbiased analysis of cell phenotypes was performed using Flojo plugins Downsample v3 and UMAP.

FACS cell sorting & TCRseq

PBMCs from three hospitalized COVID-19 patients were stained with the following antibodies to sort on the identified T cell population of interest: Live/Dead Fixable Aqua, CD3 BV786, CD4 BV605, CD8 BV650, H3K27Me3 PE, Tomm20 AF405. PBMCs were thawed as described and immediately filtered through cell strainer capped FACS tubes to avoid excessive cell clumping. Cells were stained for viability with Live/Dead Fixable Aqua and Fc Block for 10 min at room temperature followed by surface staining with CD3, CD4 and CD8 in 20% Brilliant Stain Buffer for 20 min at room temperature. Cells were washed once with PBS. Fixation/permeabilization was performed using ice cold 70% ethanol for 10 min at −20°C. Cells were washed with 2mL PBS + 0.5% BSA + 5mM EDTA and centrifuged at 2000 rpm for 5 min. ICS was performed for markers H2K27me3 and Tomm20 and cells were stained for 45 min at room temperature. Staining reactions were washed once with 2mL PBS + 0.5% BSA + 5mM EDTA and resuspended in 500uL PBS + 0.5% BSA + 5mM EDTA for sorting. $CD4^+$ and $CD8^+$ cells with $H3K27me3^+/Tomm20^+$ or $H3K27me3^-Tomm20^-$ phenotype were sorted by FACS on a Beckman Coulter MyFlo XDP Cell Sorter. Sorted cells were further processed for TCR sequencing. DNA was isolated on sorted populations using QiaAMP micro DNA kit (QIAGEN) per the manufacturer's protocol. DNA was incubated overnight in the final column step and eluted in 25uL buffer before quality was assessed via NanoDrop. TCR VbCDR3 sequencing was performed using the deep resolution Immunoseq platform (Adaptive Biotechnologies) (Carlson et al., 2013).

Single-cell RNAseq

Single cell RNA-seq libraries were prepared from viably frozen PBMCs using the 10X Chromium platform, and 5′ DGE library preparation reagents and kits according to the manufacturer's recommended protocols (10X Genomics, Pleasonton, CA). Briefly, viably frozen PBMCs were rapidly thawed at 37°C and were washed twice in DPBS to remove any dead cells and debris. Cells were counted manually with a hemocytometer and re-suspended in 0.04% BSA in DPBS to a final concentration of 1000 cells/uL. Cells and gel beads were loaded on a Chromium Next GEM Chip G to generate single cell emulsions using the 10x Chromium controller instrument with 5′ Library Kit v1.1 reagents (PN1000202, PN1000127, PN1000167, PN1000020, PN1000213). Reverse transcription, cDNA amplification, library preparation, and sample index labeling were performed according to manufacturer's protocols. Libraries were sequenced on a NovaSeq 6000 instrument to achieve a target depth of ∼50,000 reads per cell. Sequencing data were aligned and pre-processed to generate cell x gene counts matrix for each sample and also aggregate across samples using the cellranger software (v.3.1.0). These data were then imported into Seurat package (v3.1) for subsequent analysis. Data was clustered and visualized using the UMAP method. To analyze T cells, data was subsetted to include only $CD3^+$ cells. The newly subsetted data was then analyzed for differentially expressed genes between COVID-19 and healthy control samples. The gene list was then evaluated for functional enrichment of GO biological processes gene sets using PANTHER (v. 15.0) with Bonferroni correction for multiple hypothesis testing. GO terms were then condensed using ReviGO with a cutoff of 0.4. The fold enrichment determined by PANTHER was visualized. For myeloid cells, total PBMCs were first clustered using UMAP analysis. Clusters containing myeloid cells were subsetted and re-clustered. One cluster was derived predominantly from three COVID-19 patients that had high levels of $CPT1a^+VDAC^+$ myeloid cells as determined by flow cytometry. To better understand the functionality of these cells specifically, the myeloid cells from these three donors were further analyzed, revealing 4 unique clusters. Clusters 1 and 3 were analyzed for differential gene expression compared to all other myeloid cells due to the high expression of VDAC and CPT1a, matching the flow cytometry data. The gene list was then evaluated for functional enrichment using the statistical over representation with Bonferroni correction using PANTHER (v. 15.0) and GO biological processes as gene sets. GO terms were then condensed using ReviGO with a cutoff of 0.4. The fold enrichment determined by PANTHER was visualized using JMP 14 Pro.

Electron Microscopy

For transmission electron microscopy (TEM) PBMCs were thawed as described and washed once with PBS. Cells were chemically fixed as a cell pellet in 3% glutaraldehyde in 0.1M sodium phosphate buffer (pH 7.3) for 24 hours at 4°C, rinsed in 0.1M sodium phosphate buffer, and post-fixed in 1% osmium tetroxide in the same buffer for 1 hour at room temperature. The cells were dehydrated in a graded series of ethanol, transitioned with toluene, followed by infiltration and embedding in epoxy resin EPON 812 (Polysciences,Inc.). Following heat polymerization of the EPON blocks, semi-thin sections of 1000-2000nm thickness were cut and stained with 1% toluidine blue for visualization by light microscopy. Thin sections of selected areas were cut at a thickness of approximately 70-100nm (pale gold interference color) with a diamond knife (DIATOME), placed on 200 mesh copper grids, and dried at 60°C for 10 minutes. To impart electron contrast, the sections were stained with a saturated solution of uranyl acetate for 10 minutes followed by Reynold's lead citrate for 2 minutes. The sections were examined with a transmission electron microscope (JEOL JEM-1400 Plus TEM) using a lanthanum hexaboride cathode (DENKA) operating at an accelerating voltage of 60-80 keV. Images were acquired using an AMT NanoSprint12: 12 Megapixel CMOS TEM Camera (Advanced Microscopy Techniques).

Immunofluorescence

For fluorescence detection of cytochrome *c* and CD3, cells were stained as described previously (Wang et al., 2020). Briefly, PBMCs were thawed and immediately placed on a glass microscope slide using a Cytospin 2 centrifuge (Shandon). After centrifugation, the cell monolayer was fixed with 10% formalin and air-dried. Cells were permeabilized with 0.3% Triton X-100 in PBS for 10 min and blocked with 3% BSA for 45 min. Cells were then incubated with primary antibodies against cytochrome *c* (BD Biosciences, cat.no. 556432) and CD3 (Dako, cat.no. A0452) at 4°C overnight. Fluorescent staining was performed for 30 min at room temperature using highly cross-adsorbed Alexa Fluor 488 and 594 secondary antibodies (Invitrogen). Cells were washed three times in PBS after each incubation step. Following, cells were covered with mounting medium containing DAPI nuclear stain (Sigma-Aldrich) and sealed with a coverslip. Imaging was performed with a DeltaVision Elite microscope system (GE Healthcare), equipped with a Scientific CMOS camera (Chip size: 2560 × 2160 pixels), an UltraFast solid-state illumination, a 60x (N.A. 1.42) oil immersion objective and the UltimateFocus module. Single image slices were acquired and deconvolved (Softworx, Applied Precision). Image preparation and analysis was performed using Fiji (https://fiji.sc/Fiji). Intensity profiles were measured using ‘Analyze’ and ‘Plot Profile’ on contrast adjusted images. Fluorescence intensities were normalized and plotted in colors corresponding to displayed images.

For fluorescence detection of MitoTracker Deep Red Dye (ThermoFisher, M22426) PBMCs were isolated fresh and labeled with MitoTracker Deep Red Dye for 20 minutes at 37°C in complete media. Cells were then washed with PBS and stained with CD3 FITC (Clone SK7, BD Biosciences). Slides were coated with 50 μg/ml Poly-D Lysine (Sigma, P0899) and vigorously washed before addition of cells. Cells on slides were fixed with 4% paraformaldehyde (methanol free, Thermo Scientific, 28906) for 10 min before washing with PBS. Slides were then blocked in 10% goat serum (GIBCO, 16210064) followed by staining with goat anti-mouse AF488 IgG (ThermoFisher, A-11017). Slides were mounted with Slow Fade Diamond anti-fade reagent with DAPI (Invitrogen, S36964). Cells were imaged with an LSM 880-Airyscan confocal microscope equipped with a PlanApochromat 63 × /1.4 NA oil-immersion objective (Carl Zeiss). Images were processed with Zen Black software (Carl Zeiss).

In vitro T cell stimulations

PBMCs were thawed as described above, cells counted, and resuspended to $1x10^6$ cells/mL in complete media (R10; RPMI 1640/heat inactivated 10% FBS). Cells were plated in 96-well U bottom plates in the presence or absence of anti-CD3/28 stimulating antibodies (0.1mg/mL, Miltenyi), and in the presence or absence of Z-VAD-FMK (60nM, Cell Signaling Technology) or VBIT-4 (300nM, Fischer Scientific). The exact number of cells plated were stained directly *ex vivo* to calculate percent survival (number of T cells at day 0/number of T cells at 48 hours). Plates were cultured at 37°C for 48 hours and cells were stained for flow cytometry. Flow staining was performed as described above using the limited panel consisting of CD3 BV786 (BD Biosciences, cat. no. 563800), CD8 BV480 (BD Biosciences, cat. no. 566121), CD4 PE Cy5 (Biolegend, 317412), H3K27me3 PE (CST, cat. no. 40724) and VDAC1 AF532 (Abcam, cat. no. ab14734).

Feature importance and prediction analysis

Using the percentage of each cell population as features and the patients as samples, random forests (RF) were trained to classify patients into different groups using R package caret (Kuhn, 2020). The prediction performance was evaluated using the receiver operating characteristic (ROC) curve derived from leave-one-out cross-validation (LOOCV). Within each fold of LOOCV, the optimal model parameter was determined using a nested LOOCV within the training samples. Feature importance analysis was performed based on the RF models trained using all samples. A feature’s importance was calculated using the decrease of accuracy after permuting the corresponding feature in out of bag samples in the RF. Features are ordered by their importance in predicting acute COVID-19 versus Healthy controls, Severe COVID-19 versus Flu, Severe COVID-19 versus Recovered, and Severe versus Mild COVID-19. For predicting COVID-19 severity, basic clinical variables including age, sex, and BMI were also added as features to RF and feature importance analysis was rerun for predicting Severe versus Mild COVID-19. Based on the feature importance, two prediction models were rebuilt for predicting severity (Severe versus Mild COVID-19) using the top-five-ranked features (i.e., percentage of $VDAC^+CPT1a^+$ myeloid cells, PDC, and $H3K27Me3^+VDAC^+CD4^+$ cells, sex, and BMI) or the basic clinical information only (i.e., age, sex, and BMI). The two models’ performances were compared based on ROC.

QUANTIFICATION AND STATISTICAL ANALYSIS

Statistical calculations were performed in GraphPad Prism 8. Data are shown as mean ± SEM unless otherwise noted. Comparison between conditions were performed using non-parametric tests as indicated in figure legends. A p value less than 0.05 was considered significant.

Cell Reports, Volume *34*

Supplemental information

Metabolic programs define dysfunctional immune responses in severe COVID-19 patients

Elizabeth A. Thompson, Katherine Cascino, Alvaro A. Ordonez, Weiqiang Zhou, Ajay Vaghasia, Anne Hamacher-Brady, Nathan R. Brady, Im-Hong Sun, Rulin Wang, Avi Z. Rosenberg, Michael Delannoy, Richard Rothman, Katherine Fenstermacher, Lauren Sauer, Kathyrn Shaw-Saliba, Evan M. Bloch, Andrew D. Redd, Aaron A.R. Tobian, Maureen Horton, Kellie Smith, Andrew Pekosz, Franco R. D'Alessio, Srinivasan Yegnasubramanian, Hongkai Ji, Andrea L. Cox, and Jonathan D. Powell

Table S1. Flow cytometry panels and antibodies used, related to Figures 1, 2, 5 and 6.

T cell Immuno-metabolic Panel

	MARKER	FLUOROPHORE	VENDOR	CAT #	CLONE
**SURFACE**	CD3	BV786	BD Biosciences	563800	SK7
	CD8	BV480	BD Biosciences	566121	RPA-T8
	CD45RA	BV570	Biolegend	304132	HI100
	CCR7	BV650	Biolegend	353234	G043H7
	CD25	BV510	BD Biosciences	563352	M-A251
	HLA-DR	BV750	Biolegend	307672	L243
	CXCR3	BV605	Biolegend	353728	G025H7
	PD-1	BV711	Biolegend	329928	EH12.2H7
	CD4	PE-Cy5	Biolegend	317412	OKT4
	CD69	PE-Cy5.5	ThermoFisher Scientific	MHCD6918	CH/4
	KLRG1	PE-CF594	BD Biosciences	565393	2F1
	CD49a	APC	Biolegend	328314	TS2/7
	CD19	APC-Cy7	Biolegend	363010	SJ25C1
	CD56	APC-Cy7	Biolegend	362512	5.1H11
**ICS**	FoxP3	PacBlue	Biolegend	320116	206D
	Tomm20	AF405	Abcam	ab210047	EPR15581-54
	VDAC1	AF532	Abcam	ab14734	20B12AF2
	CPT1a	AF488	Abcam	ab171449	8F6AE9
	Ki67	PE-Cy7	Biolegend	350526	Ki-67
	H3K27me3	PE	CST	40724	C36B11
	HK2	AF680	Abcam	ab228819	EPR20839
	GLUT1	AF647	Abcam	ab195020	EPR3915

B cell/Myeloid Immuno-metabolic Panel

	MARKER	FLUOROPHORE	VENDOR	CAT #	CLONE
**SURFACE**	CD14	BV605	Biolegend	301834	M5E2
	CD16	BV785	Biolegend	302046	3G8
	CD33	BV570	Biolegend	303417	WM53
	CD11c	BV480	BD Biosciences	74392	B-ly6
	HLA-DR	BV750	Biolegend	307672	L243
	CCR2	BV510	Biolegend	357218	K036C2
	CD40	PacBlue	Biolegend	334320	5C3
	CD38	BV711	BD Biosciences	563965	HIT2
	CD86	BV650	Biolegend	305428	IT2.2
	IgD	BB790	BD Biosciences	Custom order	IA6-2
	CD27	PE-CF594	BD Biosciences	562297	M-T271
	CD21	PE-Cy5	BD Biosciences	551064	B-ly4
	CD138	PE-Cy5.5	Biolegend	356502	MI15
	CD15	PE-Cy7	Biolegend	301924	HI98
	LOX1	PE	Biolegend	358604	15C4
	CD123	APC	Biolegend	306012	6H6
	CD3	APC-Cy7	Biolegend	344818	SK7
	CD19	APC-Cy7	Biolegend	363010	SJ25C1
	CD56	APC-Cy7	Biolegend	362512	5.1H11
**ICS**	Tomm20	AF405	Abcam	ab210047	EPR15581-54
	VDAC1	AF532	Abcam	ab14734	20B12AF2
	CPT1a	AF488	Abcam	ab171449	8F6AE9
	HK2	AF680	Abcam	ab228819	EPR20839
	GLUT1	AF647	Abcam	ab195020	EPR3915

Table S2. Characteristics of the 38 subjects with acute COVID-19, related to Figures 1-7.

**Demographics**	
Male N (%)	19 (50)
Female N (%)	19 (50)
Mean age (range)	59.7 (20-82)
Mean BMI (range)	32.2 (17.5-51.2)
Current smoker N (%)	0 (0)
**Race and Ethnicity**	
**Race**	N (%)
Black	17 (47.5)
White	11 (28.9)
Other*	7 (18.4)
Asian	2 (5.2)
**Ethnicity**	
Hispanic/Latinx	N (%)
Yes	5 (13.2)
No	32 (86.8)
**Maximum Disease Severity****	N (%)
$MinO_2$	14 (36.7)
HFO_2	4 (10.5)
Ventilated Lived	15 (39.5)
Died	5 (13.2)
**Comorbidities**	N (%)
Hypertension	21 (55.3)
Diabetes mellitus	15 (39.5)
COPD/asthma	12 (25.0)
Coronary artery disease	2 (5.2)
HIV infection	3 (7.9)

*Most self-identified as Hispanic/Latinx.
**Maximum disease severity indicates the most severe COVID-19 disease class for the patient while under observation: $MinO_2$= no or low flow oxygen required, HFO_2= high flow oxygen required, Ventilated= patient required intubation and survived, Died = patient died (ventilated or not)

Table S3. Characteristics of study subjects. Related to Figures 1-7.

	COVID-A	Influenza	COVID-R	Acute HCV	Chronic HCV
**Demographics**					
Male N (%)	19 (50)	9 (43)	6 (60)	2 (33)	7 (70)
Female N (%)	19 (50)	12 (57)	4 (40)	4 (67)	3 (30)
Mean age (range)	59.7 (20-82)	46.4 (22-89)	47.8 (18-81)	25.8 (24-28)	30.5 (26-35)

Supplemental Figure 1

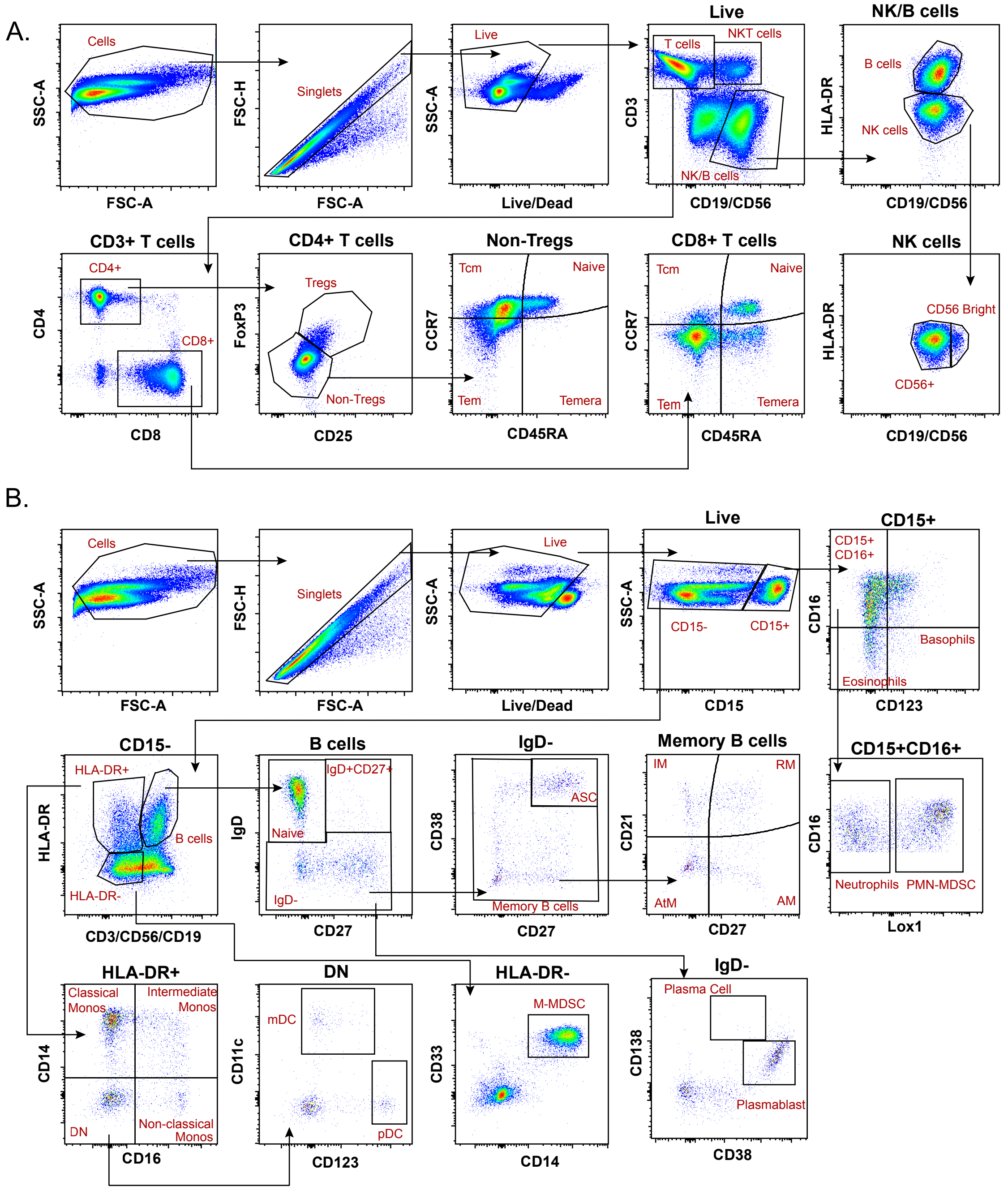

Fig. S1. Gating strategies for flow cytometry panels, related to Figures 1-2, 5-6. Representative flow plots from an acute COVID-19 subject show gating of all peripheral immune cell subsets assessed by one of two immuno-metabolic panels **(A)** T cell panel or **(B)** Myeloid and B cell panel.

Supplemental Figure 2

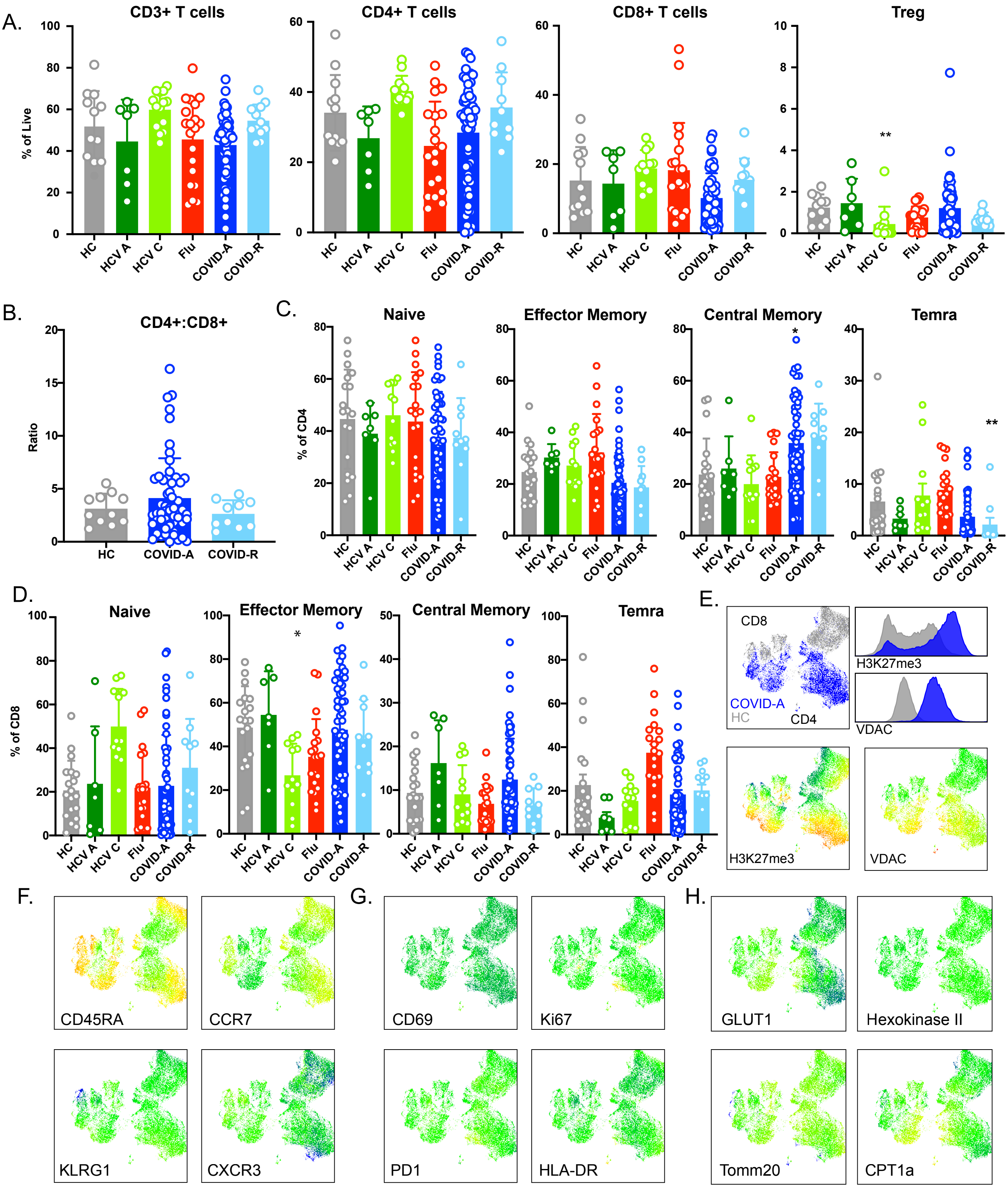

Fig. S2. Frequencies of T cell subsets and activation markers reveal few COVID19-specific differences, related to Figures 1 and 2.
(A) Frequency of indicated cell subset as percent of total live cells. Each dot represents one individual, significance tested using unpaired Kruskal-Wallis test compared to healthy control. **(B)** CD4:CD8 ratio. Each dot represents one individual, significance tested using unpaired Kruskal-Wallis test compared to healthy control. **(C)** Frequency of CD4+ and **(D)** CD8+ T cell subsets shown as percent of CD4 or CD8, respectively. Each dot represents one individual, significance tested using unpaired Kruskal-Wallis test compared to healthy control. **(E)** UMAP projection performed on a subset of COVID-A (blue) and HC subjects (grey). The two markers discovered to drive segregation of the COVID-A and HC cluster, H3K27Me3 and VDAC, are depicted as histogram overlays and MFI heatmap overlays on UMAP projection. **(F-H)** UMAP projection of MFI heatmap overlays of indicated proteins. Signifance is inidicated as compared to healthy control, *p<0.05, **p<0.01, ***p<0.001, ****p<0.0001, if no significance is indicated the test is non-significant.

Supplemental Figure 3

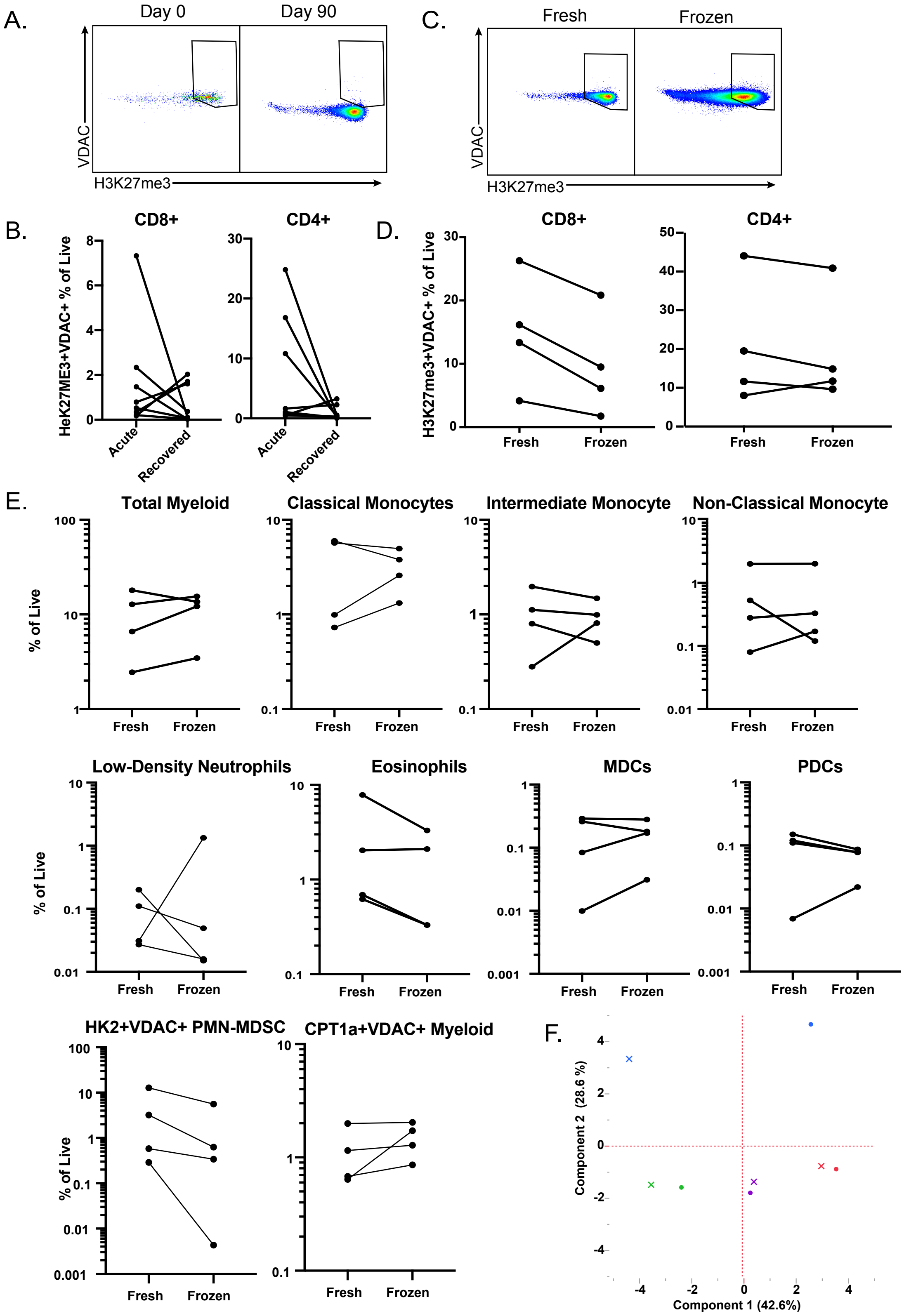

Fig. S3. Effect of cryopreservation and/or recovery on innate and adaptive compartments, related to Figures 1-2, 5-6.

(A) Representative plots show increased H3K27me3+VDAC+ CD8+ T cells in a COVID-A subject at day 0 enrollment compared to day 90 in the same subject after recovery. (B) Frequency of H3K27me3+VDAC+ T cells from 8 total COVID-A subjects with samples available at day 0 enrollment and after recovery for CD8+ T cells (left) and CD4+ T cells (right). Significance tested using Wilcoxon matched-pairs signed rank test. (C) Representative plots show increased H3K27me3+VDAC+ CD4+ T cells in a COVID-A subject with cells stained fresh compared to after cryopreservation. (D) Frequency of H3K27me3+VDAC+ T cells from 4 total COVID-A subjects tested at day 0 enrollment stained fresh or after cryopreservation for CD8+ (left) and CD4+ (right) T cells. Significance tested using Wilcoxon matched-pairs signed rank test. (E) Frequency of indicated cell subset as percent of total live cells when sample was processed fresh or after cryopreservation (frozen). Each dot represents one individual. Significance tested using Wilcoxon matched-pairs signed rank test. (F) Principal component analysis of frequencies of innate cell subsets show differences driven by patient variability and not cryopreservation.

Supplemental Figure 4

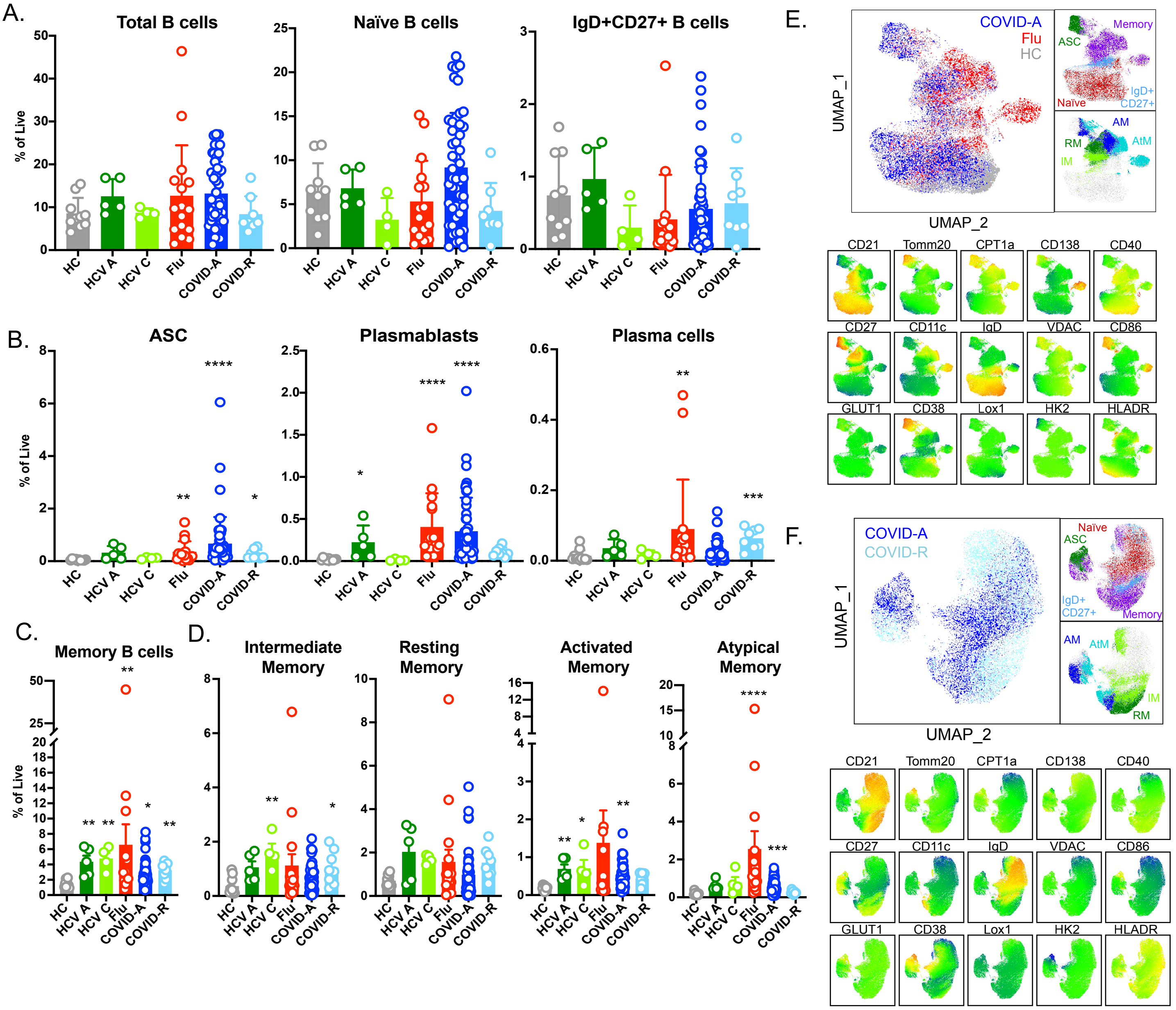

Fig. S4. B cell frequencies and phenotypes differ in the memory compartment in COVID-19, related to Figures 1 and 2.
(A-D) Frequency of indicated cell subset as percent of total live cells. Each dot represents one individual, significance tested using unpaired Kruskal-Wallis test compared to healthy control. **(E-F)** UMAP projection performed on a subset of COVID-A (blue), hospitalized Flu (red) and HC subjects (grey) **(E)** or COVID-A (blue) and COVID-R (light blue) **(F)**. Manual gating overlays on UMAP projection color code total B cell (top) and memory B cell (bottom) subsets. UMAP projection MFI heat maps of indicated proteins. Signifance is inidicated as compared to healthy control, *p<0.05, **p<0.01, ***p<0.001, ****p<0.0001, if no significance is indicated the test is non-significant.

Supplemental Figure 5

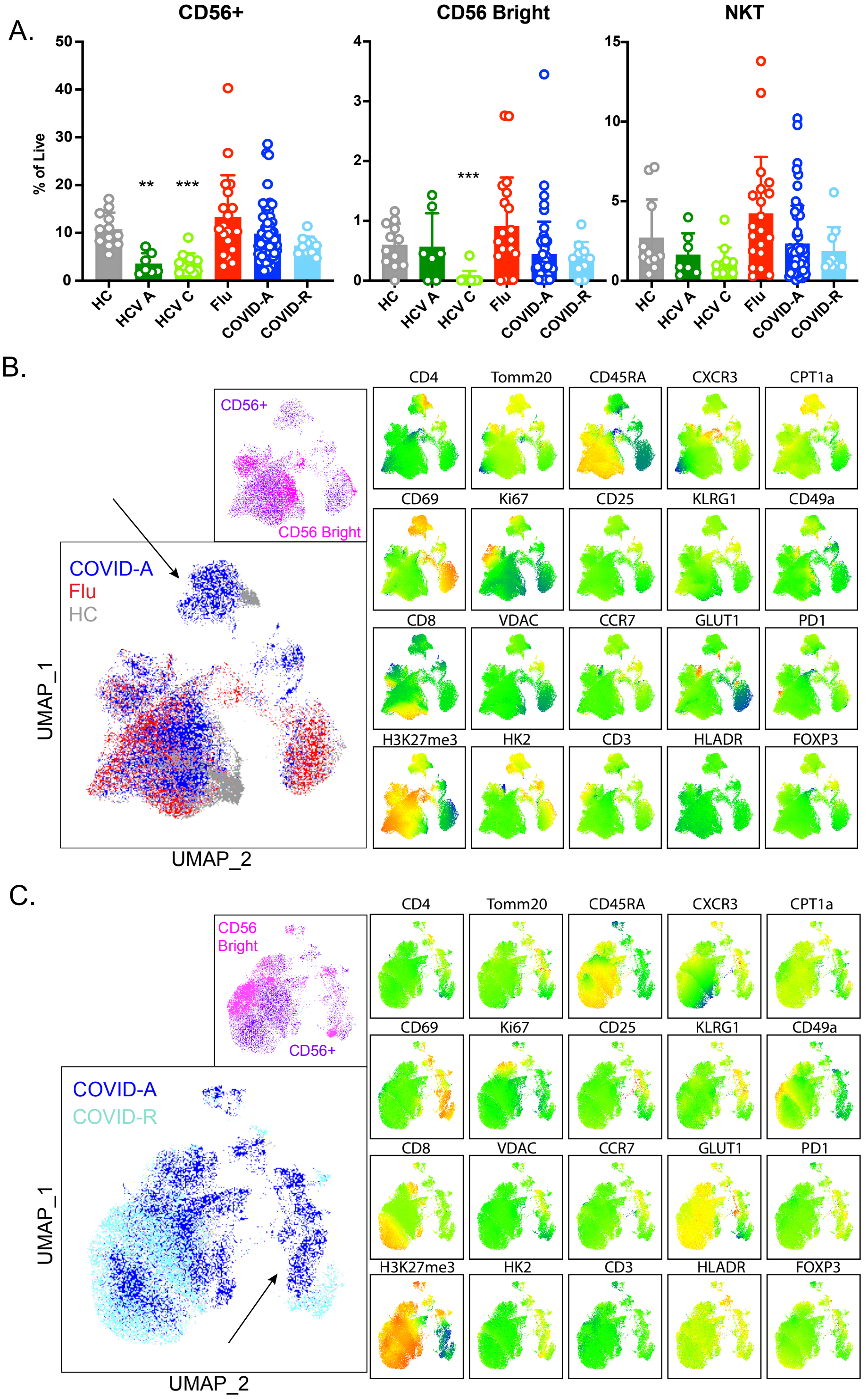

Fig. S5. Unique NK cell population in COVID-A subjects identified by high dimensional phenotyping analysis, related to Figures 1 and 2.
(A) Frequency of indicated cell subset as percent of total live cells. Each dot represents one individual, significance tested using unpaired Kruskal-Wallis test compared to healthy control. **(B)** UMAP projection of total NK cells performed on a subset of COVID-A (blue), hospitalized Flu (red) and HC subjects (grey) (left). Arrow indicates unique COVID-A specific cluster identified. Manual gating overlays on UMAP projection (top) color code CD56+ (purple) and CD56 bright (pink) cells. UMAP projection MFI heat maps of indicated proteins are shown right. **(C)** Similar analysis as in **(B)** was performed on a subset of COVID-A (blue) compared to COVID-R (light blue) subjects. Signifance is inidicated as compared to healthy control, *p<0.05, **p<0.01, ***p<0.001, ****p<0.0001, if no significance is indicated the test is non-significant.

Supplemental Figure 6

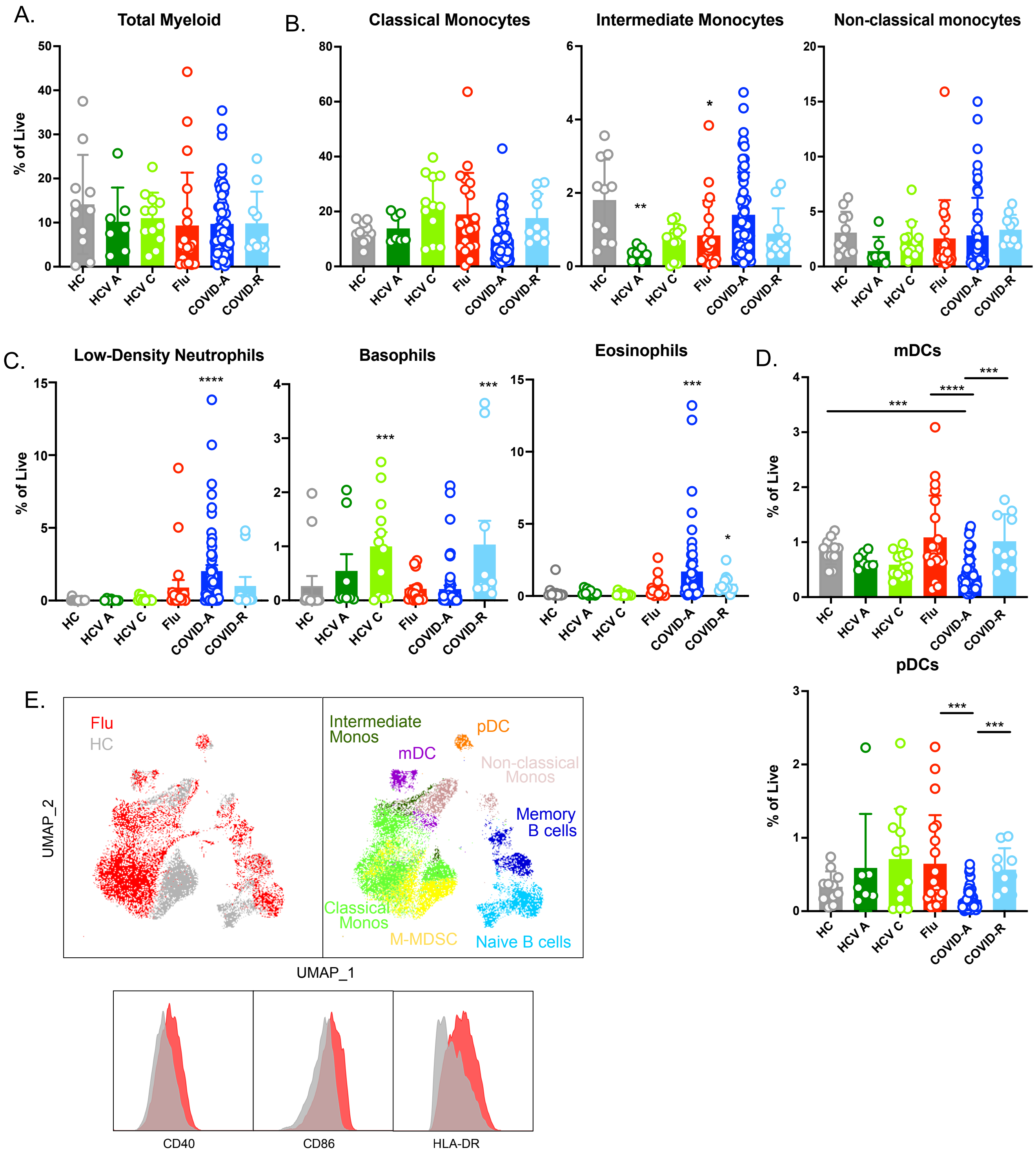

Fig. S6. Myeloid subsets in viral infections, related to Figures 5 and 6.
(A-C) Frequency of indicated cell subset as percent of total live cells. Each dot represents one individual, significance tested using unpaired Kruskal-Wallis test compared to healthy control. **(D)** Frequency of indicated cell subset as percent of total live cells. To asses how dendritic cell frequencies changed in recovery, significance was tested using unpaired Kruskal-Wallis test comparing all possible combinations. **(E)** UMAP projection of total myeloid cells performed on a subset of hospitalized Flu (red) and HC subjects (grey). Manual gating overlays on UMAP projection color code myeloid and B cell subsets in the UMAP space. MFI histogram overlays of CD14+ myeloid populations of indicated proteins for hospitalized Flu (red) and HC (grey). Signifance is inidicated as compared to healthy control (**A-C**), or between groups (**D**), *p<0.05, **p<0.01, ***p<0.001, ****p<0.0001, if no significance is indicated the test is non-significant.

Supplemental Figure 7

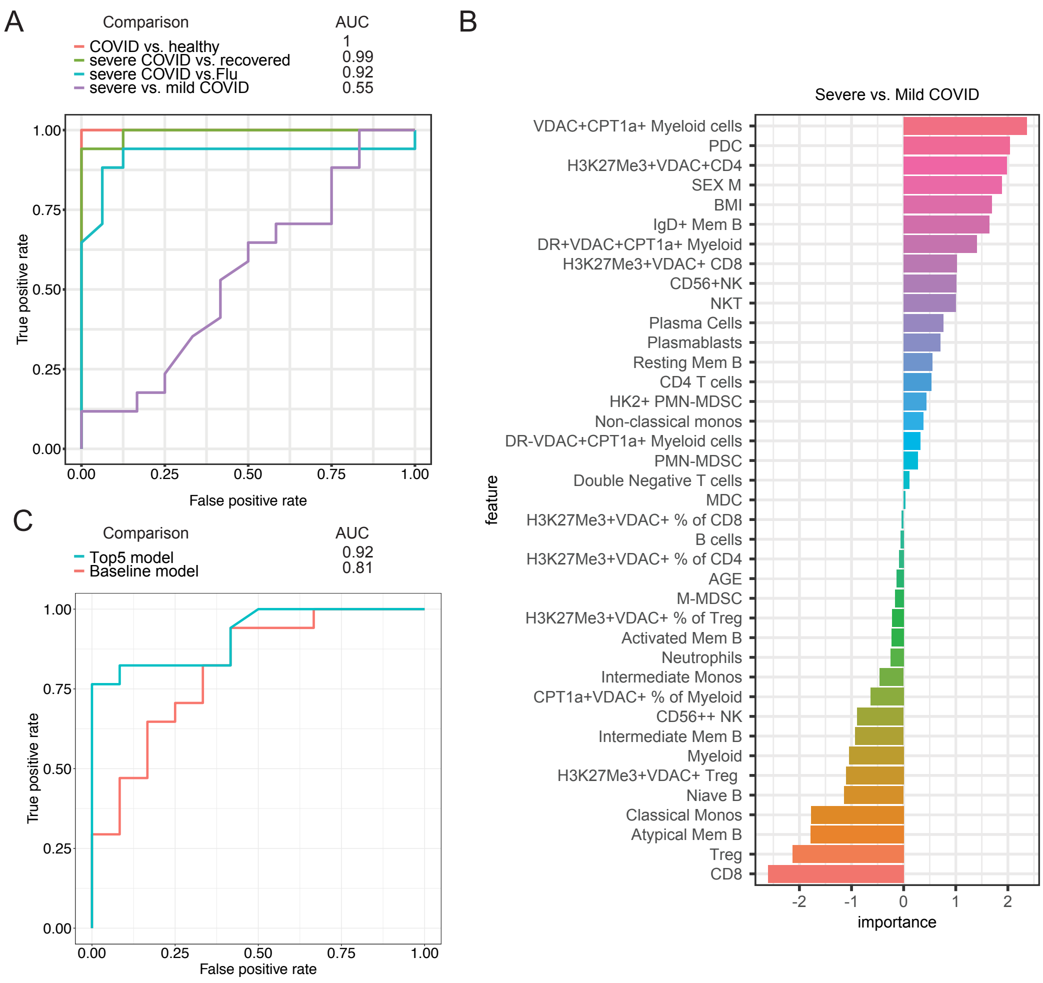

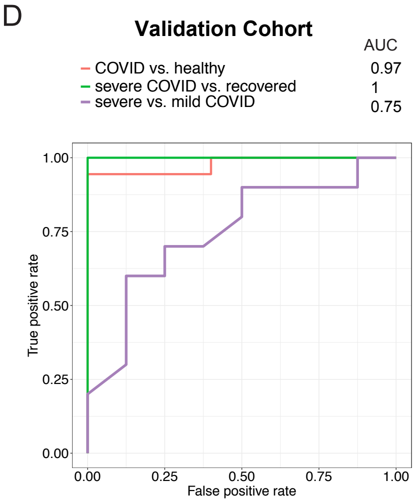

	mild vs severe COVID	severe COVID vs recovered	COVID vs HC
Accuracy	0.6667	1	0.7826
Sensitivity	0.5	1	0.7222
Specificity	0.875	1	1

Fig. S7. Metabolic profile of immune cells predicts disease status, related to Figure 7.
(A) Receiver operating characteristic (ROC) curves for predicting different groups of patients (i.e., COVID-A vs. Healthy controls, Severe COVID-A vs. COVID-R, Severe COVID-A vs. Flu, and Severe vs. Mild COVID-A). The area under the curve (AUC) is indicated. **(B)** Feature importance analysis after adding basic clinical information (i.e., age, sex, and BMI) to the RF model for classifying severe vs. mild COVID-A. **(C)** ROC curves for comparing the performance of prediction models trained using the top-five-ranked features (i.e., top5) and basic clinical information (i.e., baseline) for classifying severe vs. mild COVID-A. AUC is indicated. **D.** The data were confirmed using a validation cohort including 5 COVID-R, 10 severe COVID-A, 8 mild COVID-A and 5 healthy controls (HC).
